# Arsenic trioxide augments immunogenic cell death and induces cGAS-STING-IFN pathway activation in hepatocellular carcinoma

**DOI:** 10.1038/s41419-024-06685-8

**Published:** 2024-04-29

**Authors:** Xin Li, Yu-Fei Pan, Yi-Bin Chen, Qian-Qian Wan, Yun-Kai Lin, Tai-Yu Shang, Meng-You Xu, Tian-Yi Jiang, Meng-Miao Pei, Ye-Xiong Tan, Li-Wei Dong, Xu-Ying Wan

**Affiliations:** 1 Department of Integrated Chinese and Western Medicine, Eastern Hepatobiliary Surgery Hospital, Naval Medical University, Shanghai, China; 2 National Center for Liver Cancer, Naval Medical University, Shanghai, China; 3 Eastern Hepatobiliary Surgery Hospital, Naval Medical University, Shanghai, China; 4 School of Life Sciences, Fudan University, Shanghai, China; 5 Peking University Cancer Hospital, Beijing, China

**Keywords:** Cell death and immune response, Liver cancer

## Abstract

The treatment of hepatocellular carcinoma (HCC) is particularly challenging due to the inherent tumoral heterogeneity and easy resistance towards chemotherapy and immunotherapy. Arsenic trioxide (ATO) has emerged as a cytotoxic agent effective for treating solid tumors, including advanced HCC. However, its effectiveness in HCC treatment remains limited, and the underlying mechanisms are still uncertain. Therefore, this study aimed to characterize the effects and mechanisms of ATO in HCC. By evaluating the susceptibilities of human and murine HCC cell lines to ATO treatment, we discovered that HCC cells exhibited a range of sensitivity to ATO treatment, highlighting their inherent heterogeneity. A gene signature comprising 265 genes was identified to distinguish ATO-sensitive from ATO-insensitive cells. According to this signature, HCC patients have also been classified and exhibited differential features of ATO response. Our results showed that ATO treatment induced reactive oxygen species (ROS) accumulation and the activation of multiple cell death modalities, including necroptosis and ferroptosis, in ATO-sensitive HCC cells. Meanwhile, elevated tumoral immunogenicity was also observed in ATO-sensitive HCC cells. Similar effects were not observed in ATO-insensitive cells. We reported that ATO treatment induced mitochondrial injury and mtDNA release into the cytoplasm in ATO-sensitive HCC tumors. This subsequently activated the cGAS-STING-IFN axis, facilitating CD8^+^ T cell infiltration and activation. However, we found that the IFN pathway also induced tumoral PD-L1 expression, potentially antagonizing ATO-mediated immune attack. Additional anti-PD1 therapy promoted the anti-tumor response of ATO in ATO-sensitive HCC tumors. In summary, our data indicate that heterogeneous ATO responses exist in HCC tumors, and ATO treatment significantly induces immunogenic cell death (ICD) and activates the tumor-derived mtDNA-STING-IFN axis. These findings may offer a new perspective on the clinical treatment of HCC and warrant further study.

## Introduction

Hepatocellular carcinoma (HCC) is one of the leading causes of death from cancer worldwide [1–3]. Surgical intervention is the primary therapeutic approach for early-stage HCC. However, a substantial portion of patients are diagnosed with advanced HCC tumors, making surgical approaches impractical [4, 5]. In recent years, the immune checkpoint inhibitors (ICIs) have been approved for the treatment of unresectable HCC. However, the clinical outcomes of ICIs in HCC are not fully satisfactory. This is mainly due to the inherently ‘cold’ tumor microenvironment and the limited immunogenicity typically exhibited by HCC tumors [6–8]. Accordingly, there is an urgent need to explore novel combination therapy strategies to improve HCC immunotherapy effect.

One of the potential approaches to boost the therapeutic efficacy of ICIs is elevating the immunogenicity of HCC tumors. The immunogenic cell death (ICD), which can be induced by certain therapies, could transit cells from a non-immunogenic state to an immunogenic one [9–11]. In the context of ICD, cell death is accompanied by the generation of a spectrum of damage-related molecular patterns (DAMPs), including the translocation of calreticulin (CRT) to the cell surface and the cytosolic accumulation of mitochondrial DNA [12, 13]. Additionally, ICD leads to the release of ‘danger signals’, including high mobility group protein 1 (HMGB1) and adenosine triphosphate (ATP) molecules. These DAMPs serve as potential immunomodulators by recruiting immune cells through both endogenous and exogenous pathways [14–16]. Beyond the direct tumor-killing properties, ICD confers enduring and robust immunosurveillance capabilities against tumors [10, 17, 18]. Nevertheless, the heterogeneity of HCC patients in responses to pharmacological interventions underscores the challenge of discerning appropriate candidates for ICD-based therapeutic regimens.

Arsenic trioxide (ATO) has been approved as a primary therapeutic agent and is often used in combination with all-trans retinoic acid (ATRA) to achieve curative outcomes for acute promyelocytic leukemia (APL) [19, 20]. Furthermore, ATO has shown potential effects in the treatment of solid malignancies, including advanced HCC [21–23]. Nonetheless, the mechanisms of ATO in HCC remain incompletely elucidated.

In this study, we analyzed the heterogeneity of ATO in inhibiting the proliferation of HCC cell lines and explored the genetic characteristics of ATO-sensitive populations by combining with clinical databases and sequencing data. Meanwhile, we investigated the mechanisms of ATO in enhancing immunogenic capacities of HCC and evaluated the synergistic anti-tumor effects of combining ATO with ICIs. Our results offer promising insights into utilizing ATO-induced ICD to enhance the effectiveness of HCC immunotherapy.

## Materials and methods

### Cell lines

The Hepa1-6, Huh7, MHCC97H, Hep G2, and Hep 3B cell lines were obtained from the Shanghai Cell Bank of the Chinese Academy of Sciences. The H22 cell line is a murine hepatoma cell line derived from Balb/c mice from China Centre for Type Culture Collection (CCTCC, Wuhan, China). H22 cells were cultured in RPMI 1640 medium (L210KJ, BasalMedia) and supplemented with 10% Fetal Bovine Serum (FBS) (C04001-500, VivaCell). Hepa1-6, Huh7, MHCC97H, Hep G2, Hep 3B were supplemented with Dulbecco’s modified Eagle’s medium (L110KJ, BasalMedia) with 10% FBS. All the culture medium contained Antibiotic-Antimycotic Solution (S120JV, BasalMedia). All cells were incubated in a humidified incubator at 37 °C in an atmosphere with 5% carbon dioxide.

### Murine tumor models

All animals received humane care according to the criteria outlined in the ‘Guide for the Care and Use of Laboratory Animals’ (8th Edition), and the animal experiments protocols were approved by the Institutional Animal Care and Use Committee of the Eastern Hepatobilliary Surgery Hospital, Shanghai, China. The mouse tumor models were conducted in 6- to 8-week-old BALB/c or C57BL/6 male mice (GemPharmatech Biotechnology, Jiangsu, China). For the subcutaneous tumor models, cancer cells (1 × 10^6^) were subcutaneously injected with phosphate-buffered saline (PBS) into the right flank of mice. After 5 to 7 days, the mice were randomly grouped and treated as indicated. For the murine liver orthotopic tumors models, H22-Luc cells (3×10^5^) were resuspended in PBS and equivalently mixed with Matrigel (356237, Corning), then slowly injected into the liver lobe of Balb/c mice. After 5 days, the formation of orthotopic tumors was confirmed by bioluminescence imaging (IVIS Lumina III, PerkinElmer) and randomly grouped to receive the indicated treatments.

For the treatment of mouse tumor models, mice were intraperitoneally injected with ATO (3 mg/kg) everyday, the anti-PD-1 (BE0146, BioXcell), CD8^+^ T cell depletion antibody (Clone: 2.43, BioXCell) or IFNAR1 blocking antibody (Clone: MAR1-5A3, BioXCell) were administered three times a week at a dosage of 10 mg/kg.

The subcutaneous tumors were measured every 2 days using a caliper and the tumor volume was calculated using the formula 0.5×L×W^2^ (L for the longest diameter and W for the shortest diameter). The orthotopic tumors were monitored by bioluminescence imaging two times per week. The mice were injected intraperitoneally with 75 mg/kg D-Luciferin (ST196, Beyotime), anesthetized with isoflurane, and imaged with an IVIS lumina SERIES III imager (PerkinElmer).

### Cell viability assay

Cells were inoculated in 96-well plates (10,000 cells per well). After 24 hours, the culture medium was exchanged in the presence of varied concentrations of ATO. The cells were further incubated for the indicated time periods. Cell viabilities were detected using the CellTiter-Glo cell viability assay kit (G7570, Promega) according to the manufacturer’s instructions and measured using a SYNERGY H1 microplate reader (BioTek).

For the cell death modalities assay, the cells were preincubated with Acetylcysteine (NAC, 2 mM), Necrostatin-1 (NEC, 50 μM), Ferrostatin-1 (FER, 10 μM), Chloroquine (CQ, 10 μM) or z-VAD-FMK (z-VAD, 10 μM) for 3 h, respectively, followed by ATO treatment (0.5 or 1 μg/mL) for 48 h.

### Statistical analysis

All data presented in the figures was shown as mean ± SD. Statistical analyses were performed using GraphPad Prism and SPSS software. *p* < 0.05 was considered statistically significant. The meaning of labeling: ns, not significant; *p < 0.05; **p < 0.01; ***p < 0.001; ****p < 0.0001. The test of statistical significance was conducted through unpaired Student’s t-test, one-way ANOVA or two-way ANOVA tests as mentioned in the figure legends. The heatmaps were normalized by row/col scale using TBtools software. The Gene Set Enrichment Analysis (GSEA) was conducted to identify biological signaling pathways enriched by differentially expressed genes. Genesets from the MSigDB were used for GSEA.

## Results

### Diversity of ATO sensitivities among murine and human HCC cells

**Fig. 1:**
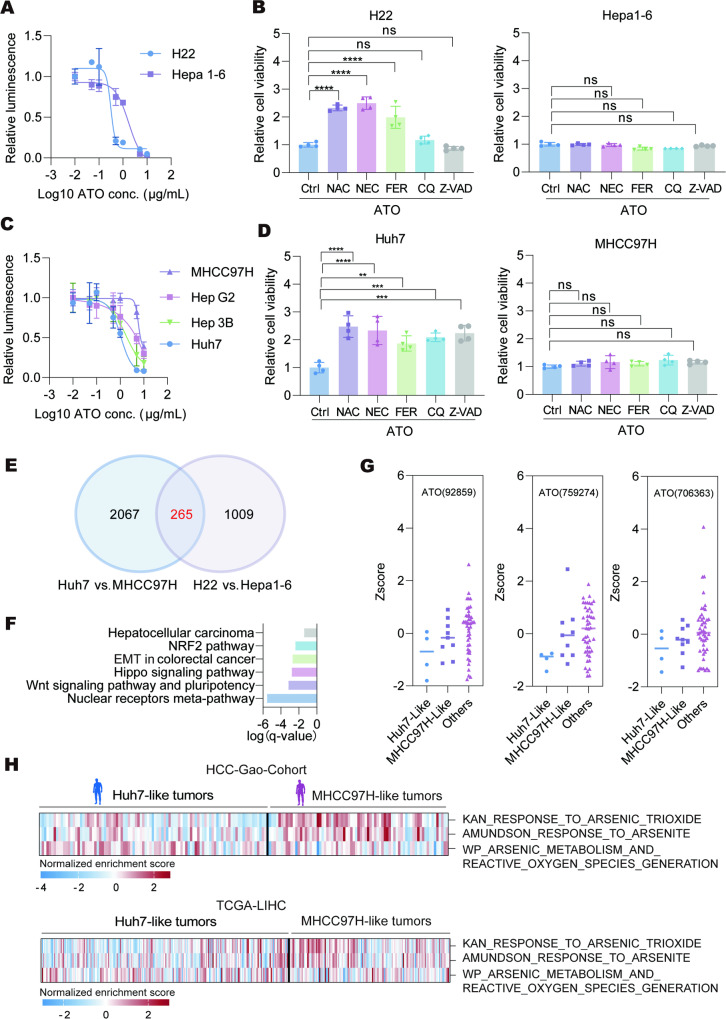
HCC cells and tumors showed diverse sensitivities to ATO treatment. **A** Relative cell viability of H22 and Hepa1-6 cells treated with the indicated concentrations of ATO for 48 h were measured by ATP-Glo assay (*n* = 3 biological replications). **B** H22 and Hepa1-6 cells were pretreated with Acetylcysteine (NAC, 2 mM), Necrostatin-1 (NEC, 50 μM), Ferrostatin-1 (FER, 10 μM), Chloroquine (CQ, 10 μM) or z-VAD-FMK (z-VAD, 10 μM) for 3 h, respectively, followed by ATO treatment (0.5 μg/mL or 1 μg/mL) for 48 h, and the relative cell viability was measured. **C** Human HCC cell lines Huh7, Hep 3B, Hep G2, and MHCC97H were treated with the indicated concentrations of ATO for 48 h, and their cell viabilities were determined (n = 3 biological replications). **D** Huh7 and MHCC97H cells were pretreated with the inhibitors as in **B** for 3 h, followed by ATO (1 μg/mL) treatment for 48 h, and the relative cell viability was measured by ATP-Glo assay. **E** The Venn-diagram of the differential expressed genes (DEGs) compared between human Huh7 to MHCC97H cells, or between murine H22 and Hepa1-6 cells with human orthologs. A total of 265 genes were commonly altered and this gene set was utilized as the features to distinguish the ‘Huh7-like’ and ‘MHCC97H-like’ signatures. **F** The Metascape analysis was conducted on the 265 genes identified in **E**, revealing significantly changed pathways. **G** Plot graphs of the indicated compounds activities (z-score) on the NCI-60 cell lines. The NCI-60 cell lines were classified according to the ‘Huh7-like’ and ‘MHCC97H-like’ signatures. **H** The ssGSEA analysis of the arsenic response associated gene signatures in HCC_Gao-cohort [24] and TCGA-LIHC cohort. The HCC samples were classified as ‘Huh7-like’ or ‘MHCC97H-like’ tumors according to the features identified in **E**. Significance was determined by one-way ANOVA in **B** and **D**. ns, not significant; **p < 0.01; ***p < 0.001; ****p < 0.0001.

To evaluate HCC cell responses to ATO, we utilized two murine HCC cell lines: H22 and Hepa1-6. The cytotoxic effects were observed in both H22 and Hepa1-6 cells following ATO treatment. Notably, H22 cells exhibited higher sensitivity to ATO (IC50: 0.3126 μg/mL) compared to Hepa1-6 cells (IC50: 1.712 μg/mL) (Fig. 1A). To investigate the cell death pathways induced by ATO, we pre-treated H22 and Hepa1-6 cells with various inhibitors, including Necrostatin-1 (NEC, for necroptosis), Ferrostatin-1 (FER, for ferroptosis), Chloroquine (CQ, for autophagy), and Z-VAD (OMe)-FMK (Z-VAD, for apoptosis). By utilizing the intracellular ATP content-based CellTiter-Glo luminescent assay, we revealed that pretreatment with NEC or FER significantly ameliorated ATO-induced cytotoxicity in H22 cells (Fig. 1B), highlighting the roles of ferroptosis and necroptosis pathways as the primary mediators of ATO-induced cell damage. Moreover, the ROS-scavenging agent N-acetyl-L-cysteine (NAC) attenuated ATO-induced inhibition of cell viability in H22 cells (Fig. 1B). However, the protective effects of the above inhibitors were not observed in Hepa1-6 cells treated with ATO (Fig. 1B), perhaps due to their relative insensitivity to ATO treatment.

We then investigated whether the diversity in ATO sensitivities also existed in human HCC cells. Among the four human HCC cell lines (Huh7, MHCC97H, Hep G2, and Hep 3B), distinct ATO sensitivities were also observed, with Huh7 displaying higher sensitivity to ATO (Fig. 1C). We then designated Huh7 as the representative ATO-sensitive cell line and MHCC97H as the ATO-insensitive counterpart for further detailed investigation. Similar to the murine HCC cells, pretreatment with NAC, NEC or FER effectively rescued ATO-induced cell viability inhibition in ATO-sensitive Huh7 cells, although exerting a less pronounced effect in MHCC97H cells (Fig. 1D), confirming the existence of heterogeneous ATO sensitivity in human HCC cells.

By analyzing of the RNA-seq data of HCC cells, we identified a gene set comprising 265 genes that are commonly altered between ATO-sensitive and -insensitive cells in both human and mouse HCC (Fig. 1E, Supplementary Table 1). Pathway analysis revealed enrichment of the NRF2, Wnt, and Hippo signaling pathways according to the 265 genes (Fig. 1F). We then conducted a comprehensive analysis utilizing the NCI-60 database from the National Cancer Institute and divided it into ‘Huh7-like’ and ‘MHCC97H-like’ clusters based on the characteristics of the 265-gene set. We observed a higher sensitivity to ATO in the ‘Huh7-like’ cluster in the NCI-60 database (Fig. 1G). We also analyzed the clinical HCC cohorts, including the HCC-Gao-Cohort [24] and TCGA-LIHC, and effectively matched the patients with our ‘Huh7-like’ and ‘MHCC97H-like’ features (Fig. 1H). We found that the ‘response to arsenic trioxide’ signatures were significantly enriched in ‘MHCC97H-like’ patients, whereas the ‘arsenic metabolism and ROS generation’ signatures were enriched in ‘Huh7-like’ tumors (Fig. 1H), suggesting the diversity of ATO sensitives in HCC patients.

### ATO induces immunogenic cell death in HCC cells

**Fig. 2:**
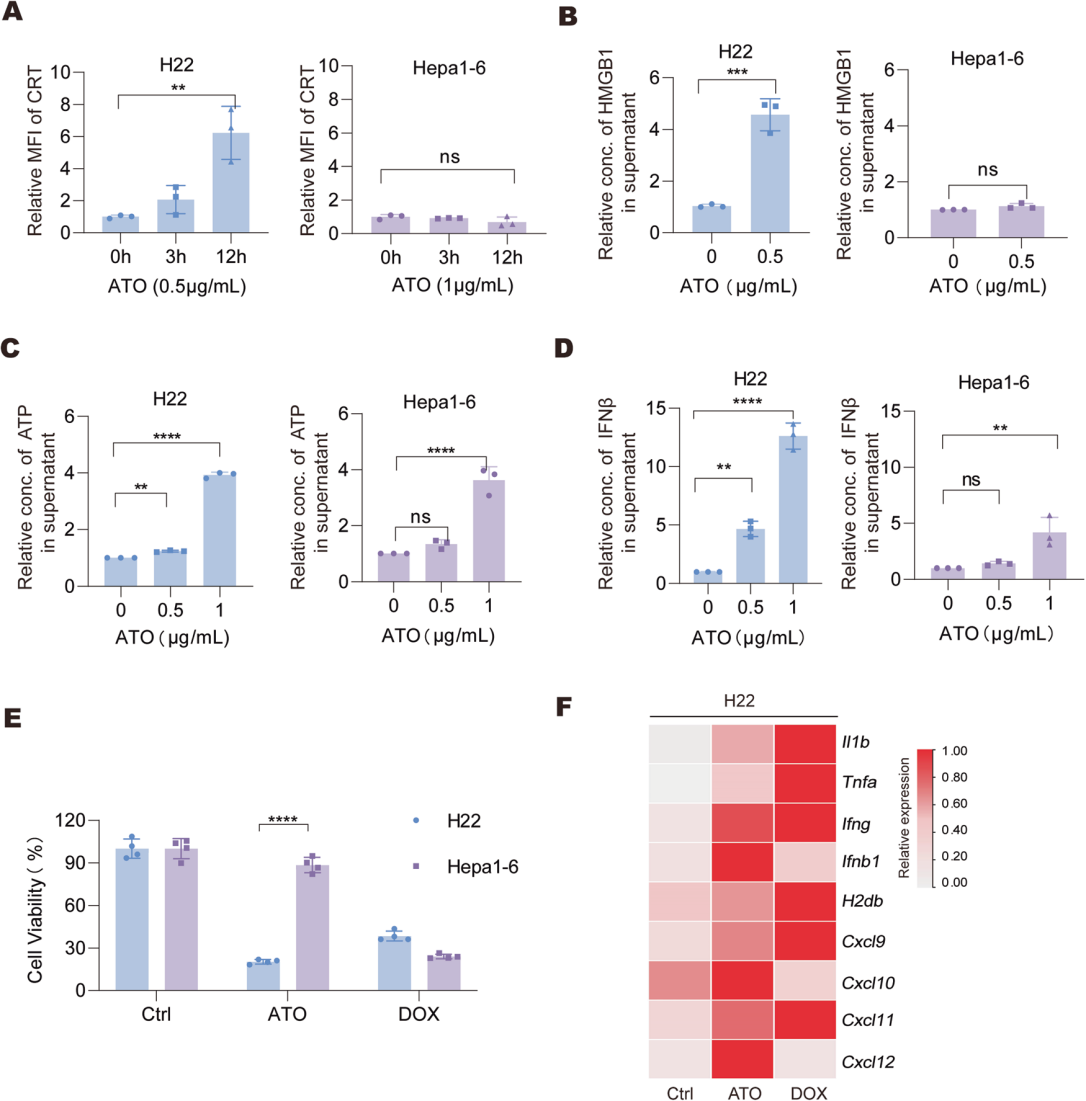
ATO treatment leads to immunogenic cell death (ICD) in ATO-sensitive HCC cells. **A** Murine HCC cells were treated with ATO (H22, 0.5 μg/mL; Hepa1-6, 1.0 μg/mL) for 3 or 12 h. The cell surface calreticulin (CRT) levels were measured by flow cytometry. **B** The relative concentrations of HMGB1 in the supernatant of H22 and Hepa1-6 cells treated with ATO (0.5 μg/mL) for 20 h were quantified by ELISA. **C**, **D** The supernatants of H22 and Hepa1-6 cells treated with ATO for 20 h were collected, and the ATP levels were measured by ATP-Glo kit in **C**. The relative IFNβ contents were quantified by ELISA in **D**. **E** H22 and Hepa1-6 cells were treated with ATO (0.5 μg/mL) or doxorubicin (DOX, 1 μM) for 20 h, and the relative cell viabilities were measured. **F** The changes of *Il1b*, *Tnfa*, *Ifng*, *Ifnb1*, *H2db*, *Cxcl9*, *Cxcl10*, *Cxcl11*, and *Cxcl12* in H22 cells treated with ATO and DOX were detected by qPCR. Significance was determined by one-way ANOVA in **A,** **C,** and **D**, Student’s t-test in **B** and **E**. ns not significant; *p < 0.05; **p < 0.01; ***p < 0.001; ****p < 0.0001.

The multiple cell death mechanisms induced by ATO prompted us to investigate whether ATO treatment could enhance the immunogenicity of HCC. We observed rapid translocation of CRT onto the cell membrane, a key indicator of ICD, in H22 cells after ATO treatment (Fig. 2A). By contrast, no significant changes in CRT surface exposure were found in ATO-insensitive Hepa1-6 cells (Fig. 2A). Furthermore, the release of HMGB1, ATP, and IFNβ were significantly increased in H22 cells in response to ATO treatment, while only slight changes were observed in ATO-treated Hepa1-6 cells (Fig. 2B–D). Compared with the classical ICD-inducer doxorubicin (DOX), ATO exhibited stronger cytotoxicity in H22 cells than in Hepa1-6 cells (Fig. 2E). The transcripts of cytokines (including *Il1b* and *Tnfa*), interferons (including *Ifng* and *Ifnb1*), and various chemokines (including *Cxcl9, Cxcl10, Cxcl11*, and *Cxcl12*) in H22 cells treated with ATO were elevated, reaching levels comparable to those induced by DOX (Fig. 2F). No similar result was observed in Hepa1-6 cells treated with ATO (Supplementary Fig. 1A). Collectively, these results demonstrate that ATO treatment can induce ICD-like phenotypes in ATO-sensitive HCC cells.

### ATO enhances anti-tumor immunity in HCC

**Fig. 3:**
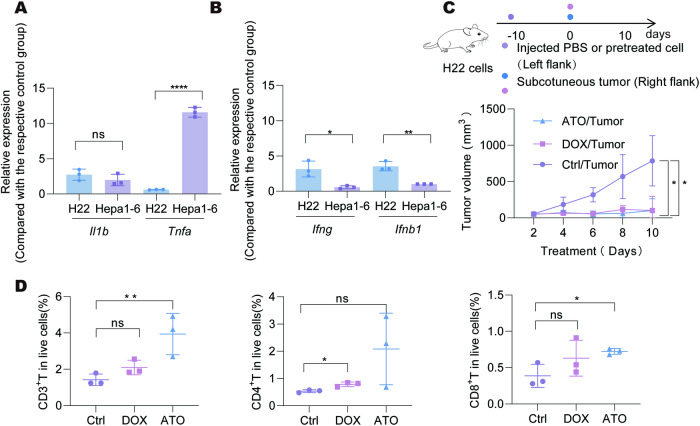
ATO treatment elicits prophylactic activities. **A, B** H22 and Hepa1-6 cells were treated with ATO (0.5 μg/1 μg) for 20 h. The relative expression of *Il1b* and *Tnfa* (**A)** as well as *Ifng* and *Ifnb1* (**B)** were detected by qPCR and shown as the fold changes comparing with their basal levels respectively. **C** Mice were vaccinated with ATO or Dox-pretreated dying tumor H22 cells and rechallenged with the corresponding live tumor cells 10 days later. Tumor growth kinetics were monitored for each group (*n* = 5–6 mice/per group). **D** The remaining tumor tissues as in **C** were collected and the infiltrated CD3^+^ T cells and their subpopulations CD4^+^ T and CD8^+^ T cells were analyzed by flow cytometry. Significance was determined by Student’s t-test in **A** and **B**, two-way ANOVA in **C** and one-way ANOVA in **D**. DOX, doxorubicin. ns, not significant; * *p* < 0.05; ** *p* < 0.01; ****p < 0.0001.

We attempted to investigate whether ATO could function as an immunogenic agent for the treatment of liver cancer. ATO treatment increased *Il1b* expression in both H22 and Hepa1-6 cells, while the expression alteration of *Tnfa* was more significant in Hepa1-6 cells (Fig. 3A). However, elevated interferon-associated factors and chemokines were observed in H22 cells compared to Hepa1-6 cells (Fig. 3B and Supplementary Fig. 1A). To assess the immune-priming role of ATO-induced whole-cell vaccine, we conducted an experiment where Balb/c mice were immunized with syngeneic H22 cells pretreated with either ATO or DOX for 10 days, followed by the injection of live H22 tumor cells at the opposite side of the immunized mice. The sites that received ATO- or DOX-preconditioned cells did not form tumor masses. We found that mice immunized with an ATO-induced vaccine were protected from tumor rechallenge (Fig. 3C). Similar anti-tumor effects were observed in the DOX-induced vaccine (Fig. 3C). Increased accumulation of CD3^+^ T cells, including both CD8^+^ T cells and CD4^+^ T cells, were found in the rechallenged tumors of mice receiving the ATO-induced vaccine (Fig. 3D). These findings suggest that ATO treatment promotes the recruitment of T cells to tumor sites, contributing to its anti-tumor effects, confirming the potential role of ATO as an anti-tumor immunogenic inducer.

### ATO induces mitochondrial damage in HCC cells

**Fig. 4:**
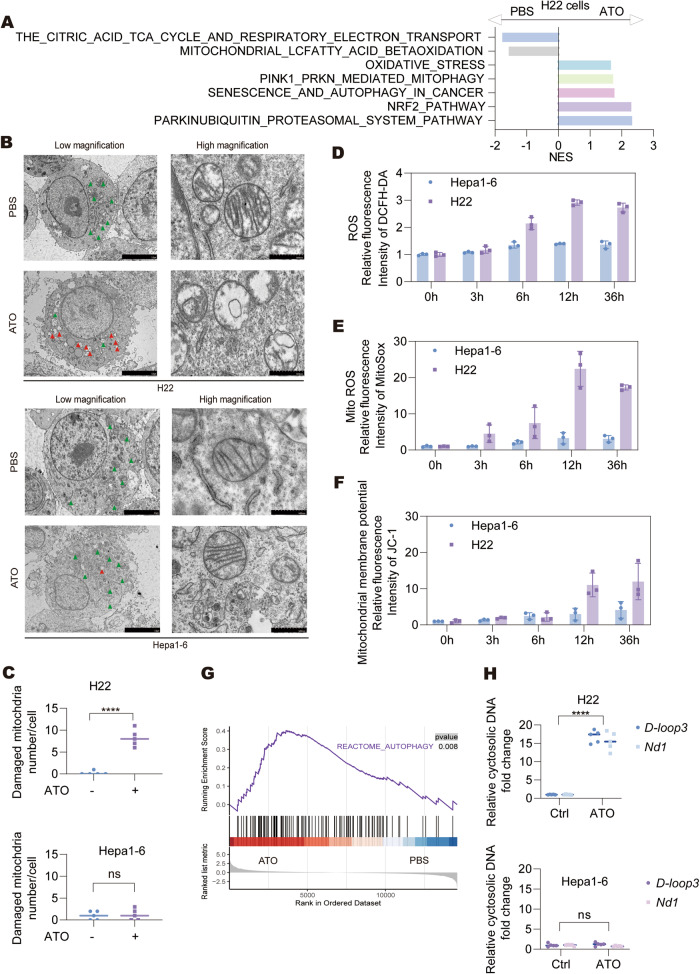
ATO treatment induces mitochondrial damage in HCC cells. **A** GSEA analysis of the transcriptomes of H22 cells treated with PBS or ATO (0.5 μg/mL) for 20 h. The top gene signatures with FDR < 0.05 were listed. **B** The electron microscopy morphology of H22 cells treated with ATO (0.5 μg/mL, upper panel) and Hepa1-6 cells treated with ATO (1.0 μg/mL, lower panel) for 16 h. The mitochondria were indicated by arrows and their high magnifications were shown. The images were the representatives of at least 5 view fields. **C** The number of damaged mitochondria per cell as indicated in **B** was counted and analyzed. **D**–**F** H22 cells and Hepa1-6 cells were treated with ATO (0.5 μg/mL and 1.0 μg/mL, respectively) for the indicated times. The relative changes of intracellular ROS levels (**D)**, mitochondrial ROS (**E)** and mitochondrial membrane potential (**F)** were measured by flow cytometry using the indicated kits. **G** GSEA analysis of ‘REACTOME_AUTOPHAGY’ signature in H22 cells treated with ATO (0.5 μg/mL) or PBS for 20 h. **H** The cytoplasmic mtDNA was extracted from H22 and Hepa1-6 cells treated with PBS or ATO for 20 h and the expression of *D-loop3* and *Nd1* were detected by qPCR. Significance was determined by Student’s t-test in **C** and **H**. ns not significant; ****p < 0.0001.

To further elucidate the mechanism of ATO-induced cell death, KEGG analysis was performed based on the RNA-seq data of H22 cells treated with PBS or ATO (Supplementary Table 2). This revealed the enrichment of pathways related to mitochondrial damage and autophagy in ATO-treated H22 cells (Fig. 4A). Electron microscopy further illustrated increasing mitochondrial damage in the cytoplasm of H22 cells exposed to ATO (Fig. 4B, C), characterized by alterations in mitochondrial crests and mitochondrial edema. Notably, the extent of damaged mitochondria in Hepa1-6 cells treated with ATO was lower than that observed in H22 cells.

ROS is one of the causes in mitochondrial damage. We employed DCFH-DA to assess the ROS levels in H22 and Hepa1-6 cells after ATO treatment. We observed a significant ROS accumulation in H22 cells, but not in Hepa1-6 cells (Fig. 4D). Meanwhile, the mitochondrial ROS levels detected by MitoSOX and the mitochondrial membrane depolarization detected by JC-1 also showed significant alterations in ATO-treated H22 cells compared to Hepa1-6 cells (Fig. 4E, F). Interestingly, the number of mitochondria, as assessed using MitoTracker, remained largely unaffected (Supplementary Fig. 2A). In summary, our investigations confirmed the occurrence of mitochondrial damage as a result of ATO treatment.

We observed gradual increases in LC3 lipidation and reductions in p62 protein levels, as well as enrichment of autophagy-associated genes in the ATO-treated H22 cells (Fig. 4G, and Supplementary Fig. 2B), indicating the activation of autophagy-related processes. Notably, we found that ROS accumulation played an important role in ATO-mediated autophagy induction (Supplementary Fig. 2C, D). By employing the MT-mKeima-Red mPrkn vectors, it was revealed that ATO treatment induced mitophagy in H22 cells in a time-dependent manner, as shown by the increased percentages of cells with dysfunctional mitochondria localization in an acidic environment (pH<4) (Supplementary Fig. 2E). These results were consistent with the enriched ‘PINK1-PRKN mediated mitophagy’ signature in ATO-treated H22 cells (Supplementary Fig. 2F). Meanwhile, no obvious mitophagy was found in Hepa1-6 cells treated with ATO (Supplementary Fig. 2E).

Mitochondrial damage leads to the release of cellular components, including mtDNA, a potent DAMP that triggers inflammatory responses through pattern recognition receptors (PRRs). Quantitative PCR analysis showed a notable rise in cytoplasmic mtDNA in H22 cells following ATO treatment, whereas no similar change was noted in Hepa1-6 cells (Fig. 4H). At the same time, ATO treatment had little impact on genomic DNA (gDNA) levels in either H22 or Hepa1-6 cells (Supplementary Fig. 2G).

Taken together, these data demonstrate ATO-induced mitochondrial damage and the release of mtDNA into the cytoplasm in ATO-sensitive cells.

### ATO-induced mitochondrial damage activates the cGAS-STING pathway in HCC cells

**Fig. 5:**
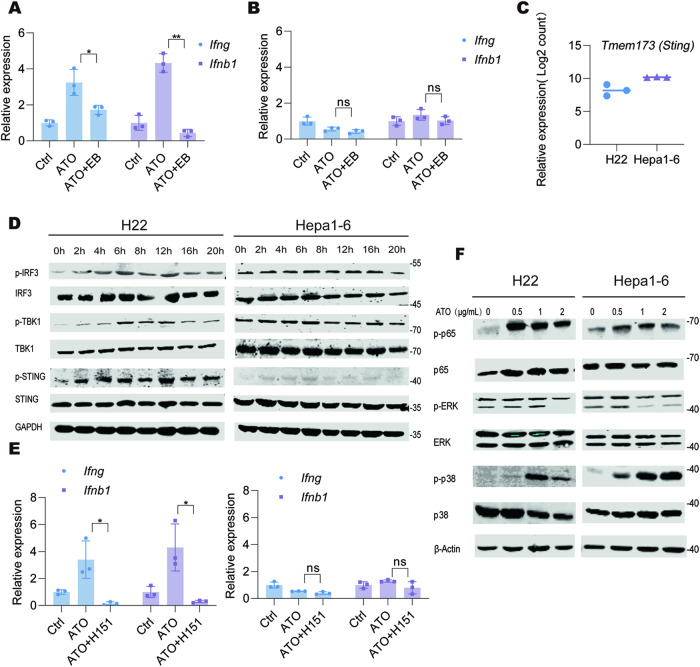
ATO-induced mitochondrial injury contributes to cGAS-STING activation. **A**, **B** H22 (**A)** and Hepa1-6 (**B)** cells were cultured with or without ethyl bromide (EB, 120 ng/mL) for 3 days, followed by ATO (0.5 μg/mL) or PBS treatment for another 20 h. The transcripts of *Ifng* and *Ifnb1* were quantified. **C** The transcripts of *Tmem173 (Sting)* was quantified by Log2 count in RNA-seq. **D** H22 and Hepa1-6 cells were treated with ATO (0.5 μg/mL and 1.0 μg/mL, respectively) for the indicated times and Western blot analysis was performed to assess the activities of the STING-IRF-3 pathway using the indicated antibodies. GAPDH was measured as a loading control. The images were the representatives of three independent experiments. **E** H22 and Hepa1-6 cells were treated with ATO in the presence of H151 (500 nM) or DMSO for 20 h. The transcripts of *Ifng* and *Ifnb1* were quantified by qPCR and shown as the fold changes comparing with their basal levels respectively. **F** Western blot analysis of the phosphorylation levels of p65, ERK and p38 proteins in H22 and Hepa1-6 cells treated with indicated concentrations of ATO for 20 h. β-Actin was measured as a loading control. The images were the representatives of three independent experiments. Significance was determined by one-way ANOVA in **A**, **B** and **E**. ns, not significant; * *p* < 0.05; ** *p* < 0.01.

We hypothesized that ATO-induced release of mtDNA might play a role in activating anti-tumor immunity. To substantiate this finding, we treated cells with ethidium bromide, a substance that depletes mtDNA within cells. This treatment resulted in a decrease in the expression of various chemokines and cytokines in H22 cells after ATO exposure (Fig. 5A). The similar trend was not observed in the ATO-insensitive Hepa1-6 cells (Fig. 5B), suggesting that mtDNA could serve as a crucial trigger for immune modulation.

Subsequently, we sought to identify the specific PRRs responsible for the inflammatory activation induced by ATO. We found that the level of *Tmem173* (*Sting*) transcripts in both H22 and Hepa1-6 cells exceeded those of other endogenous nucleic acid sensing genes (Fig. 5C and Supplementary Fig. 3A). Higher *STING* expression was also found in human HCC cell lines (Supplementary Fig. 3B). The cGAS/STING pathway is identified as a key sensor for detecting inappropriate cytosolic DNA [25]. Our experiments showed that ATO treatment in H22 cells induced STING phosphorylation in a time-dependent manner, alone with gradual phosphorylation of TBK1 and IRF-3 (Fig. 5D). Furthermore, the expression of IRF-3 downstream targets, including the type I interferons, were also increased in response to ATO, while the STING inhibitor H151 reduced their expression (Fig. 5E). These results demonstrate that ATO treatment activates the cGAS/STING pathway and promotes interferon expression. Activation of the cGAS/STING pathway was not observed in Hepa1-6 cells, unlike in H22 cells, in response to ATO (Fig. 5D, E).

The NF-κB and MAPK pathways are also regulated by cGAS/STING activation [26]. ATO treatment significantly increased the phosphorylation of NF-κB p65 in H22 cells, indicating the activation of the NF-κB pathway (Fig. 5F). Interestingly, unlike the cGAS/STING pathway, we found that the NF-κB and MAPK pathways were also activated by ATO in Hepa1-6 cells (Fig. 5F). Consistent with this result, the NF-κB targeted genes (*Il1b* and *Tnfa*) were dramatically increased in ATO-treated Hepa1-6 cells (Fig. 3A, B).

### ATO treatment elevated tumoral PD-L1 expression

**Fig. 6:**
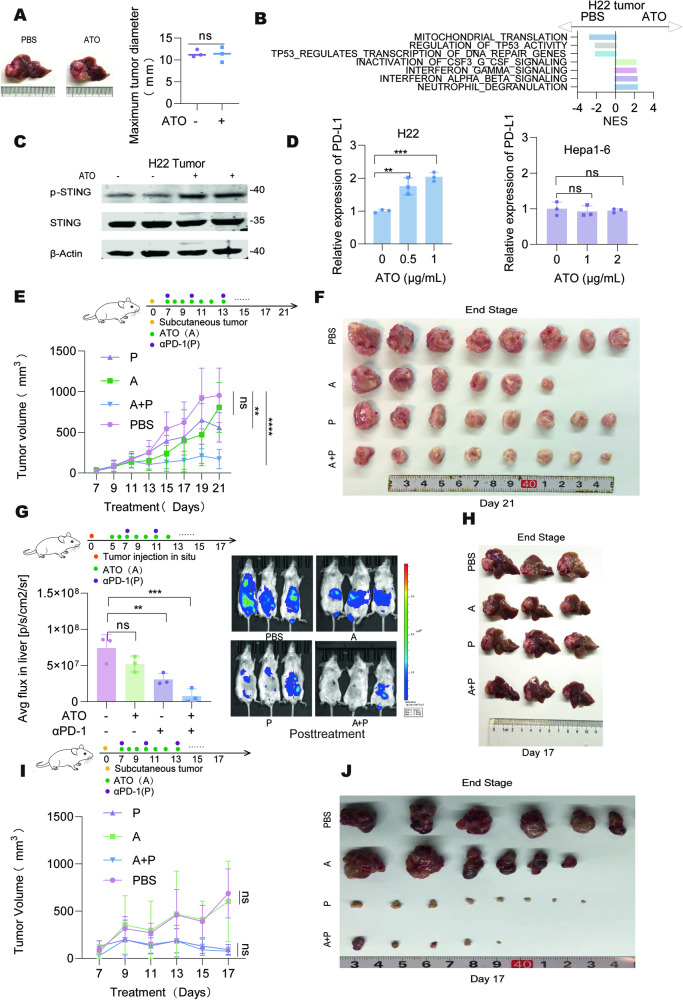
Synergetic anti-tumor effect of PD1-blocking antibody in combination with ATO in ATO-sensitive HCC cells. **A** Murine H22 cells were orthotopically implanted into Balb/c mice. After 7 days, the mice were treated with PBS or ATO (3 mg/kg) every two days, and the tumors were collected and measured on day 17. The representative liver images were shown (*n* = 5 mice/group). **B** The transcriptomes of the tumor tissues shown in **A** were assessed by RNA-seq and GSEA analysis were performed. The top significantly altered pathways with FDR < 0.05 were listed. **C** Western blot analysis of STING phosphorylation levels in tumor tissues shown in **A** and the representative images were presented. **D** The relative expression of PD-L1 in H22 and Hepa1-6 cells treated with ATO for 20 h was detected by flow cytometry. **E**, **F** H22 cells were subcutaneously inoculated into Balb/c mice. After 7 days, the mice were randomly grouped and treated with PBS, ATO (A, 3 mg/kg), αPD-1 (P, 10 mg/kg) or ATO combined with αPD-1, respectively. The tumor tissues were collected on day 21 with tumor growth quantified (**E)**, and the macroscopy at the end stage was shown in **F** (*n* = 6-8 mice/group). **G** H22-Luc cells were orthotopically implanted into Balb/c mice. After 5 days, the specified mice were pretreated with ATO (A, 3 mg/kg) twice. On day 7, tumor-bearing mice received the treatments as described in **E**. Tumor growth was monitored by bioluminescence imaging. **H** Macroscopic images of orthotopic tumors from **G**. **I**, **J** Hepa1-6 cells were subcutaneously inoculated into C57BL/6 mice. After 7 days, the mice were randomly grouped and treated with the treatments as described in **E**. The tumor tissues were collected on day 17 with tumor growth quantified (**I)**, and the macroscopy at the end stage was shown in **J** (*n* = 6-8 mice/group). Significance was determined by two-way ANOVA in **E** and **I**, one-way ANOVA in **D** and **G**. ns, not significant; *p < 0.05; **p < 0.01; ***p < 0.001; ****p < 0.0001.

The above results demonstrated the effective cytotoxic activity of ATO in certain HCC cells in vitro. Therefore, we speculate that ATO could serve as a potent anti-tumor agent in vivo. Surprisingly, we found that administration of ATO only slightly inhibited the growth of liver orthotopic H22 tumors (Fig. 6A), indicating the presence of additional mechanisms that counteract the cytotoxic effects of ATO. RNA-seq analysis highlighted mitochondrial damage and interferon-related pathways in tumors post-ATO treatment (Fig. 6B, Supplementary Fig. 4A and Supplementary Table 3). STING phosphorylation was notably elevated in ATO-treated tumor samples compared to those treated with PBS (Fig. 6C), confirming the immune modulation effect of ATO in H22 cells. We speculated that the alteration of immune checkpoints induced by ATO might be responsible for the resistance of the drug’s anti-tumoral effect. Indeed, PD-L1 level significantly increased in ATO-treated H22 cells (Fig. 6D). After removal of mitochondrial DNA or treatment with H151, ATO no longer upregulated *PD-L1* expression in H22 cells (Supplementary Fig. 5 A). We also observed a strong positive correlation between *cGAS/STING* and *CD274* levels in the TCGA-LIHC dataset (Supplementary Fig. 5B). These findings indicate to a complex interaction between ATO-induced STING activation and immune checkpoint regulation in tumor cells.

### Combining ATO with ICIs promotes antitumor immune response in ATO-responsive HCC tumors

We hypothesized that blocking the ATO-induced PD-L1 expression might enhance the anti-tumor effects of ATO in HCC. While anti-PD-1 antibody monotherapy had limited inhibitory effects on the tumor growth of H22 tumors, combining it with ATO significantly enhanced the therapeutic efficacy (Fig. 6E, F and Supplementary Fig. 6A). Additionally, ATO-treated H22 tumors exhibited elevated PD-L1 expression and increased CD8^+^ T cell infiltration in vivo compared to PBS-treated tumors (Supplementary Fig. 7A). For real-time monitoring of tumor growth, we generated H22-Luc cells and implanted them into the livers of mice, facilitating bioluminescence imaging. In this model, the combination of ATO with anti-PD-1 markedly inhibited tumor progression (Fig. 6G, H and Supplementary Fig. 6B). Similarly, ATO-treated H22 tumors exhibited elevated PD-L1 expression and increased CD8^+^ T cell infiltration in vivo compared to PBS-treated tumors (Supplementary Fig. 7B). In both models, the combination of ATO with anti-PD-1 enhanced the infiltration of CD8^+^ T cells compared to either ATO or PD-1 monotherapy (Supplementary Fig. 7C, D). We also used ATO-insensitive Hepa1-6 cells for subcutaneous tumor implantation. It was observed that while this tumor was sensitive to anti-PD-1 therapy, the addition of ATO did not enhance the effectiveness of anti-PD-1 monotherapy (Fig. 6I-J).

**Fig. 7:**
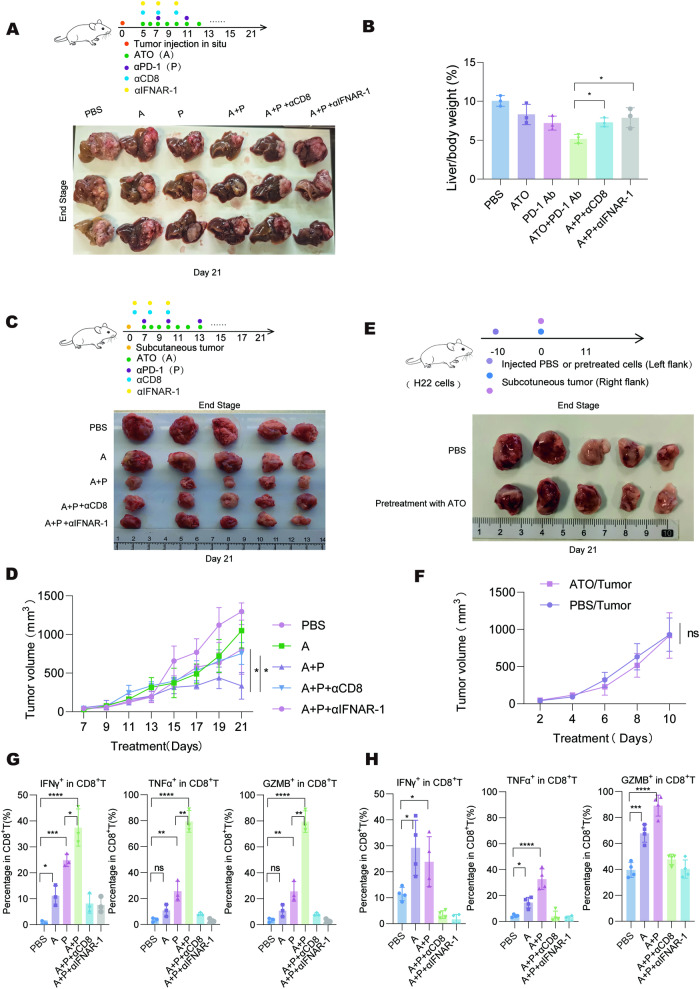
ATO promoted CD8^+^T cell infiltration and activation in tumor. **A**, **B** H22-Luc cells were orthotopically implanted into Balb/c mice. After 5 days, the mice were randomly assigned to various treatment groups, including PBS, ATO (A, 3 mg/kg), αPD-1 (P, 10 mg/kg), ATO + αPD-1, ATO + αPD-1 + αCD8 (10 mg/kg) and ATO + αPD-1 + αIFNAR-1 (10 mg/kg). The tumor tissues were collected on day 21. The administration process of the treatments and tumor macroscopy was shown in **A**. The tumor growth was quantified in **B**. **C**, **D** H22 cells were subcutaneously inoculated into Balb/c mice. After 5 days, the mice were randomly grouped and treated separately with the treatment doses described in **A**. The tumor tissues were collected on day 21. The administration process of the treatments and tumor macroscopy was shown in **C**. The tumor growth was quantified in **D**. **E**, **F** Nude mice were vaccinated with ATO-pretreated dying H22 cells and rechallenged with the corresponding live tumor cells 10 days later. Tumor growth kinetics were monitored for each group (*n* = 5–6 mice/per group). The tumor macroscopy was shown in **E** and the tumor growth was quantified in **F**. **G** Tumor infiltration of IFN-γ, TNFα, and Granzyme B (GZMB) in H22 orthotopic tumors was measured by flow cytometry. **H** Tumor infiltration of IFN-γ, TNFα and GZMB in H22 subcutaneous tumors was measured by flow cytometry. Significance was determined by two-way ANOVA in **D** and **F**, one-way ANOVA in **B**, **G** and **H**. ns, not significant; *p < 0.05; **p < 0.01; ***p < 0.001; ****p < 0.0001.

Subsequently, the activation status of CD8^+^ T cells in tumors was assessed. Synergistic anti-tumor effects were observed when ATO was combined with PD-1 antibody in both the subcutaneous and orthotopic inoculation models (Fig. 7A–D). Treatment with the PD-1 antibody alone could reactivate the tumor-infiltrated CD8^+^ T cells in H22 tumors by increasing the proportions of IFNγ^+^, TNFα^+^ and Granzyme B^+^ (GZMB^+^) cells within the CD8^+^ T cell population (Fig. 7G, H). However, it did not enhance the infiltration of CD8^+^ T cells into the tumor region (Supplementary Fig. 8A–C). By contrast, ATO treatment promoted the accumulation of tumor-infiltrating CD8^+^ T cells and increased the proportion of IFNγ^+^ cells in the CD8^+^ T cell population (Fig. 7G, H and Supplementary Fig. 8B, C). Notably, the combination of ATO with PD-1 antibody treatment significantly activated CD8^+^ T cells compared to tumors treated with either ATO or PD-1 antibody alone, demonstrating the potential synergistic effect of this combination therapy on enhancing immune response against tumors.

We then evaluated whether CD8^+^ T cells and the cGAS/STING/IFN pathway are required for the immune-priming function of ATO in HCC tumors. Blocking either CD8^+^ T cell or the IFN pathway significantly impeded the anti-tumor effects of ATO when combined with PD-1 antibody. This blockade inhibited the capacity of ATO to facilitate the infiltration and activation of CD8^+^ T cells in both subcutaneous and orthotopic inoculation models (Fig. 7A–D, G, H, and Supplementary Fig. 8B, C). Meanwhile, the ATO-induced H22 vaccine did not affect H22 rechallenge in immune-deficient Balb/c-nude mice (Fig. 7E, F). Taken together, these results demonstrate that both CD8^+^ T cells and IFN pathways play essential roles in the in vivo immune-priming function of ATO on tumors.

## Discussion

Although ATO, either alone or in conjunction with ATRA, has revolutionized the treatment of APL, its therapeutic effect in solid tumors remains limited [22, 27]. Our study has verified the heterogeneity of ATO in inhibiting the proliferation of HCC cell lines and summarized the genetic characteristics of ATO-sensitive populations. Notably, we have underscored the potential significance of cellular oxidative stress as a determinant influencing HCC’s responsiveness to ATO.

**Fig. 8:**
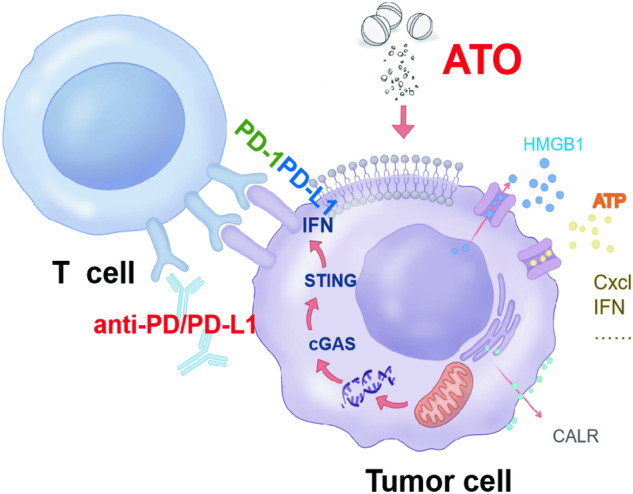
The pattern diagram of the synergistic effects of ATO in combination with PD-1 antibody.

There is growing evidences suggesting that therapy-induced ICD can effectively act as ‘in situ vaccines’ to enhance tumor immune surveillance [28–30]. In our investigation, pretreatment with ATO not only effectively eliminated tumor cells in vitro but also significantly enhanced the antigenicity and adjuvant properties of these cells. Notably, when these treated tumor cells were administered as a vaccine in mice, they demonstrated both prophylactic and therapeutic efficacy, resulting in a substantial reduction in tumor growth. This phenomenon was characterized by a cascade of molecular processes, including the rapid ectopic translocation of CRT and the release of ATP and HMGB1. The goal of tumor elimination is accomplished through the release of IFN, which triggers the secretion of cytokines and chemokines within the tumor microenvironment, leading to the recruitment of CD8^+^ T cells (Fig. 8).

Chemotherapy has a significant impact on mitochondrial biology [31–33]. We have found that ATO has an affinity for mitochondria, leading to the generation of mtROS that disrupts mitochondrial function. The mtDNA is a potent DAMP that triggers inflammatory responses through various PRRs [34, 35]. Thus, mitochondrial damage can fine-tune the availability of inflammatory molecules. The stimulator of interferon genes (STING), also known as TMEM173, serves as a PRR responsible for detecting mtDNA [36–39]. ATO can serve as an immunostimulant by inducing mitochondrial damage and the release of mtDNA, thereby activating the cGAS/STING/IFN cascade.

Conceptually, human cancers can be categorized into two main groups: ‘hot’ tumors and ‘cold’ tumors. The ‘cold’ tumors have limited immune cell infiltration and are generally resistant to ICIs [40, 41]. Various therapeutic strategies are currently employed in combination with ICIs to boost immune cell recruitment and convert ‘cold’ tumors into ‘hot’ ones [27, 42–44]. Remarkably, ATO has shown the ability to induce immunogenic cell death in tumor cells while concurrently increasing the expression of PD-L1, a known immune checkpoint protein, through a mechanism dependent on mtDNA-STING signaling (Fig. 8). The synergistic application of ATO and ICIs demonstrates significant improvement in the responsiveness of HCC to ICIs. We have also proven that the effectiveness of this combined therapy relies on the activity of CD8^+^ T cells and the induction of type I interferon.

In summary, we have identified diverse sensitivities of HCC in response to ATO treatment and demonstrated that ATO plays a pivotal role in promoting mitochondrial damage in HCC cells. This effect promotes mtDNA-dependent STING pathway, leading to recruitment and activation of CD8^+^ T cells. Meanwhile, this pathway promotes the expression of PD-L1 on tumor cells to evade immune surveillance. Our findings support the feasibility of conducting additional clinical trials to explore the combination of ATO and ICIs to enhance the therapeutic effects in HCC.

### Supplementary information

Supplementary Information

Supplementary Table 1-3

Full and uncropped Western blots

## Data Availability

Data are available upon reasonable request. All data and material generated in this study are available upon request from the corresponding author.
