# Supplementary material for: Arsenic trioxide augments immunogenic cell death and induces cGAS-STING-IFN pathway activation in hepatocellular carcinoma
Source: Cell Death Dis. 2024 Apr 29;15(4):300. doi: 10.1038/s41419-024-06685-8 (PMC11058202; doi:10.1038/s41419-024-06685-8)
Supplement: Supplementary file 1 — Supplementary Information [file 41419_2024_6685_MOESM1_ESM.pdf]

Supplementary Information

Arsenic Trioxide Augments Immunogenic Cell Death and Induces

cGAS-STING-IFN Pathway Activation in Hepatocellular Carcinoma

Xin Li *et al.*

*Corresponding author. Email: wanxuying@126.com

This PDF file includes:

Supplementary materials and methods

Supplementary Figure 1-8

Supplementary Table 4-6

Other Supplementary Materials for this manuscript include:

Supplementary Table 1-3

Full and uncropped Western blots

Supplementary materials and methods

Detection of immunogenic cell death associated factors

The cell surface CRT was analyzed by flow cytometry. Cells were incubated with mouse anti-CRT (ab142141, Abcam) primary antibody at 4°C for 30 min. The cells were then washed twice and labeled with secondary antibody at 4°C for 15 min. The content of supernatant HMGB1 was measured by an ELISA kit (SEKM-0145, Solarbio). The supernatant IFNβ was measured by an ELISA kit (EK2236, MULTI SCIENCES). The supernatant ATP was measured by CellTiter-Glo Luminescent Cell Viability Assay Kit (G7570, Promega).

Cytoplasmic DNA extraction

The cells were incubated in digitalin buffer (150 mM NaCl, 50 mM HEPES, 50 μg/mL digitalin) at 4°C for 10 min. The homogenate was then centrifuged at 980 g at 4°C for 4 minutes. The supernatant was centrifuged again at 17,000 g at 4°C for 10 min to remove any remaining cell debris. The DNA in the supernatant was extracted using TIANamp DNA Kit (DP304-03, Tiangen) and the contents were determined by NanoDrop (Thermo Fisher). A Total of 10 ng DNA per sample was taken, and the mitochondrial DNA was measured using the primers listed in the supplementary material (Supplemental table 4).

RNA collection, cDNA synthesis, and real-time PCR analysis

Total RNA was extracted from indicated cells using TRIzol (15596018, Invitrogen), and cDNA synthesis was performed using a reverse transcription kit (R223-01, Vazyme). The qRT-PCR was conducted using FastStart Universal SYBR Green Master (4913850001, Roche)

in LightCycler 480II (Roche). All primer sequences are listed in Supplemental table 4.

RNA-seq analysis

Total RNA (1 μg) was extracted from cell samples, followed by library preparation according to Illumina's standard instruction using the VAHTS Universal V6 RNA-seq Library Prep Kit. Agilent 4200 Bioanalyzer was employed to evaluate the concentration and size distribution of the cDNA library before sequencing on an Illumina NovaSeq6000. The protocol of high-throughput sequencing strictly followed the manufacturer's instructions. The raw reads were filtered by Seqtk before being mapped to the genome using Hisat2 (version 2.0.4). Gene fragments were counted using StringTie (v1.3.3b), followed by trimmed mean of M-values (TMM) normalization. Significant differentially expressed genes (DEGs) were identified as those with a False Discovery Rate (FDR) value below the threshold ($q < 0.05$) and fold-change > 2 using edgeR software.

Western blot

Whole-cell lysate was prepared using RIPA buffer (P0013B, Beyotime) and centrifuged at 12000 g for 15 min. Protein concentrations were measured using the Pierce BCA Protein Assay Kit (23209, Thermo Scientific). The protein levels of interest were detected using specific primary antibodies (Supplemental table 5) and measured using an Odyssey fluorescence scanner (Li-COR).

Electron microscopy

Cell precipitates were obtained via centrifugation, and the medium was subsequently removed. The samples were suspended and fixed by an electron microscope fixative (Servicebio,

G1124) at 4°C for 3 h. After fixation, the supernatant was discarded, and the samples were rinsed three times with 0.1 M PBS. The samples were then embedded in a pre-heated 1% agarose solution. A 1% osmic acid solution prepared with 0.1 M PBS was employed for fixation at room temperature for 2 h, away from light. The samples were rinsed three times with PBS, and dehydrate at room temperature using gradient ethanol and acetone. This process culminated in penetration with a 1:1 mixture of epoxy resin and acetone for 3 h, followed by overnight permeation with a 2:1 mixture. The samples were further embedded in pure epoxy resin and polymerized at 60°C in a drying oven for 48 h. The resin block was finely sectioned into slices measuring 60-80 nanometers using an ultra-thin slicing machine. The slices were then transferred onto 150 mesh copper grids coated with formvar film. Staining was performed with a 2% uranium acetate-saturated alcohol solution in the absence of light for 8 min, followed by a triple rinse with 70% alcohol. Subsequently, the sections were stained with a 2.6% lead citrate solution under carbon dioxide avoidance for 8 minutes and washed three times with ddH_2O. The sections were gently dried with filter paper and placed into a copper mesh box, allowing them to dry overnight at room temperature. The prepared samples were analyzed by a transmission electron microscope (HITACHI, HT7800/HT7700).

ROS detection and mitochondrial status analysis

Cells were incubated with 10 mM DCFH-DA (S0033S, Beyotime) for 30 min for intracellular ROS measurement. Mitochondrial Superoxide analysis was carried out by incubating cells at 37°C for 10 min in complete media containing 200 nM MitoSOX Red Mitochondrial Superoxide Indicator dye (40778ES50, YEASEN). For mitochondrial membrane polarization

analysis, cells were incubated in complete medium containing 5 mg/mL JC-1 (40705ES03, YEASEN) dye at 37℃ for 15 min. The mitochondria were labeled with MitoTrackerRed staining (40741ES50, YEASEN).

Mitochondrial DNA depletion

For mitochondrial DNA depletion, cells were cultured in the presence of Ethidium bromide (120 ng/mL) for 3 days. The cell DNA was extracted and the mtDNA was measured to confirm its depletion.

Mitophagy

The MT-mKeima-Red plasmid (AM-V0251, MBL) and the pcDNA3.1A-mParkin plasmid were co-transfected into cells at a ratio of 2:1 using Lipo8000 transfection reagents (C0533, Beyotime) following the manufacturer's protocol. After 36 h, the cells received the indicated treatment and were analyzed by flow cytometry. The healthy mitochondria were determined by high mKeima pH7 fluorescence (Ex: 488 nm, Em: 586 nm) and low mKeima pH4 fluorescence (Ex: 561 nm, Em: 586 nm). Cells with high mKeima pH4 and low mKeima pH7 were noted as undergoing mitophagy.

Histological examination

Paraffin-embedded tumor tissues were cut into 5-mm sections and stained with hematoxylin and eosin (H&E) for analysis of morphological changes. Immunohistochemistry was performed with the indicated antibodies (Supplemental table 5). The percent area of positive staining was determined using ImageJ software. For histological quantification, the average number of positive-staining cells in 5 or 6 high-power fields from one tissue section was

calculated.

Tissue preparation and flow cytometry analysis

The tumor tissues were minced and enzymatically digested in serum-free medium supplemented with 1.0 mg/mL collagenase type IV (Sigma) and 30 U/mL DNase type I (Sigma) for 60 min at 37°C. The cells were then filtered through 70 μm cell strainers (Miltenyi Biotec) and washed with PBS. Red blood cells were removed by red blood cell lysis buffer (Dakewe). After washed once with PBS, the cells were resuspended in cell staining buffer (00-4222-26, eBioscience). After blocking Fc-receptors by TruStain FcX (101320, BioLegend), cells were stained with indicated antibodies (Supplemental table 5). Zombie Aqua (423102, BioLegend) was used to identify living or dead cells. $CD4^+$ T cells were identified as $CD45^+CD3^+CD19^-CD4^+CD8^-$; $CD8^+$ T cells were identified as $CD45^+CD3^+CD19^-CD4^-CD8^+$. For detection of IFN-γ, TNFα and Granzyme B, cells were prestimulated with activation cocktails (423303, Dakewe) and PMA/Ionomycin. The cells were then incubated at 37°C for 6 h. Flow cytometry was performed on a 4-laser BD LSRFortessa X-20 (BD Biosciences) using FACSDiva software, and the data was subsequently analyzed with FlowJo VX software.

Supplementary Figures

Supplementary Figure 1. ATO-induced chemokines expression in HCC cells.

(A) H22 and Hepa1-6 cells were treated with ATO (H22, 0.5 μg/mL; Hepa1-6, 1 μg/mL) for 20 h as in Figure **2F**, the relative expression of *Cxcl9, Cxcl10, Cxcl11,* and *Cxcl12* in cells was detected by qPCR and presented as the fold changes comparing with their basal levels respectively. Significance was determined by Student's t-test. ns, not significant. * $p < 0.05$; ** $p < 0.01$.

Supplementary Figure 2. ATO-induced mitochondrial damage and mitophagy in ATO-sensitive cells.

(A) H22 and Hepa1-6 cells were treated with ATO (0.5 μg/mL and 1.0 μg/mL, respectively) for the indicated times. The relative changes of mitochondrial numbers were determined by MitoTracker staining. **(B)** H22 and Hepa1-6 cells were treated with the indicated concentrations of ATO for 20 h. Western blot analysis was performed to assess the levels of LC3, P62 and BECLIN-1. β-Actin was measured as a loading control. The images were the representatives of three independent experiments. **(C)** H22 cells were pretreated with Acetylcysteine (NAC), followed by the indicated concentrations of ATO for 20 h. Western blot analysis was performed to assess the expression levels of LC3 and P62. β-Actin was measured as a loading control. The images were the representatives of three independent experiments. **(D)** H22 cells were pretreated with H151, followed by the indicated concentrations of ATO for 20 h. Western blot analysis was performed to assess the levels of LC3 and P62. β-Actin was measured as a loading control. The images were the

representatives of three independent experiments. **(E)** The MT-mKeima-Red (KR) together with mPrkn plasmids were transfected into H22 and Hepa1-6 cells to monitor mitopahgy. The cells were then treated with ATO for the indicated times, harvested, and analyzed by flow cytometry. The values shown indicate the percentage of cells with high levels of mitophagy (KR pH<4). **(F)** GSEA analysis of 'REACTOME_PINK1_PRKN_MEDIATED _MITOPHAGY' signature in H22 cells treated with ATO (0.5 μg/mL) or PBS for 20 h. **(G)** The gDNA was extracted from H22 and Hepa1-6 cells treated as described in Figure **4H**. The expression of *Gapdh* was detected by qPCR as an indicator of gDNA.

Supplementary Figure 3. Expression levels of pattern-recognized receptors in HCC cells.

(A) The transcripts of *Tlrs*, *Nods* and *Tmem173* in H22 and Hepa1-6 cells. **(B)** The transcriptional levels of *TLRs*, *NODs* and *STING1* in 25 HCC cell lines obtained from the Depmap database.

Supplementary Figure 4. ATO induced significant transcriptome changes in tumors.

(A) PCA analysis of the transcripts from liver orthotopic tumors treated with ATO (3 mg/kg) or PBS.

Supplementary Figure 5. ATO induced PD-L1 expression.

(A) H22 cells were treated with EB (120 ng/mL) or H151 (500 nM), and followed by ATO for 20 h. The mRNA expression of PD-L1 was quantified by qPCR and the relative fold changes compared with its control were shown. **(B)** The Pearson correlation analysis of the expression of *STING*, *MB21D1* (*cGAS*) with *CD274* in TCGA-LIHC cohort. ** $p < 0.01$; *** $p < 0.001$.

Supplementary Figure 6. The anti-PD1 therapy promoted ATO-mediated anti-tumor effects.

(A) The tumor weights of the subcutaneous H22 tumors as described in Figure **6E**. **(B)** H22-Luc cells were orthotopically implanted into Balb/c mice as described in Figure **6G**. The tumors were monitored by bioluminescence imaging 5 days before the subsequent therapy. Significance was determined by one-way ANOVA in **(A)** and **(B)**. ns, not significant. * $p < 0.05$; *** $p < 0.001$.

Supplementary Figure 7. PD1-blocking antibody in combination with ATO promoted $CD8^+$ T cells infiltration in tumors.

(A) Immunohistochemical analysis of $CD8^+$ T cell infiltration and PD-L1 expression of subcutaneous tumors from Figure **6E**. The images were the representatives of at least 5 view fields. The quantification of the areas of CD8α and PD-L1 positive staining was shown in the right panel. **(B)** Immunohistochemical analysis of $CD8^+$ T cell infiltration and PD-L1 expression in liver orthotopic H22 tumors from Figure **6H**. The images were the representatives of at least 5 view fields. The quantification of the areas of CD8α and PD-L1 positive staining was shown in the right panel. **(C)** Tumor infiltration of $CD8^+$ T cells in H22 orthotopic tumors measured by flow cytometry. **(D)** Tumor infiltration of $CD8^+$ T cells in H22 subcutaneous tumors measured by flow cytometry. Significance was determined by Student's t-test in **(A)**, **(B)**, **(C)** and **(D)**. * $p < 0.05$; ** $p < 0.01$.

Supplementary Figure 8. $CD8^+$ T cells are required for the anti-tumor effect of ATO

(A) Gating strategies for flow cytometry data. **(B)** The proportions of $CD8^+$ T cells in $CD45^+$

cells in H22-Luc orthotopic tumors with different treatments (as described in Figure **7A**) was measured by flow cytometry. **(C)** The proportions of $CD8^+$ T cells in $CD45^+$ cells in H22 subcutaneous tumors with different treatments (as described in Figure **7C**) was measured by flow cytometry.

Supplementary Figure 1

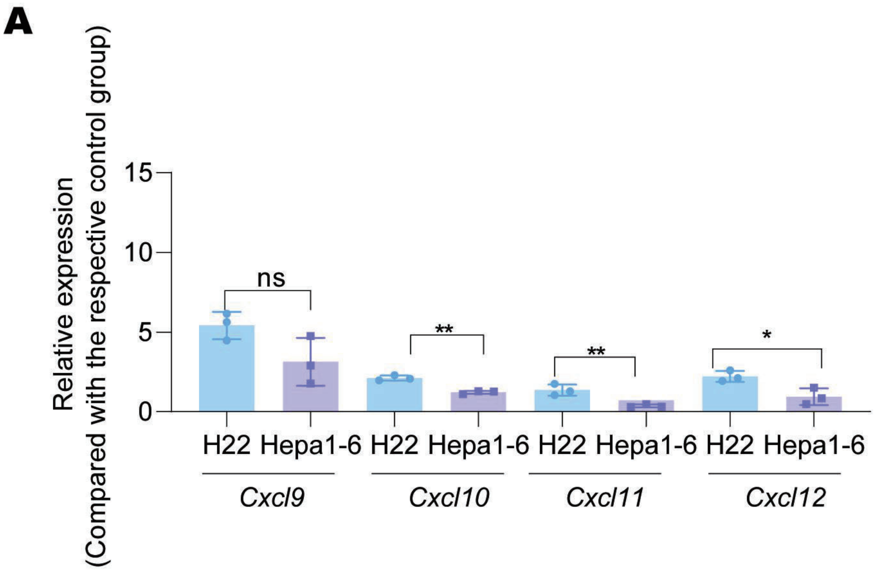

Supplementary Figure 2

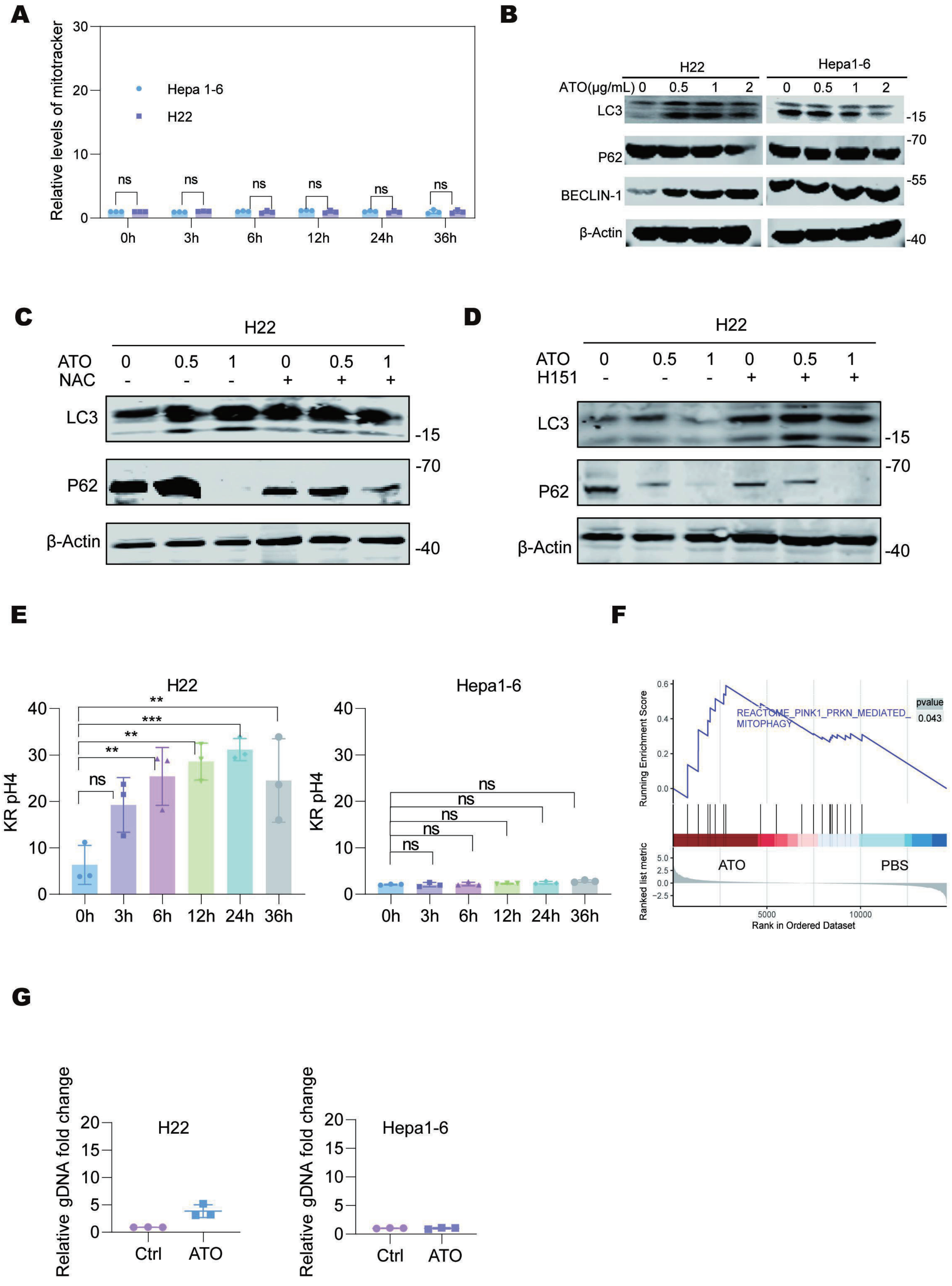

Supplementary Figure 3

A

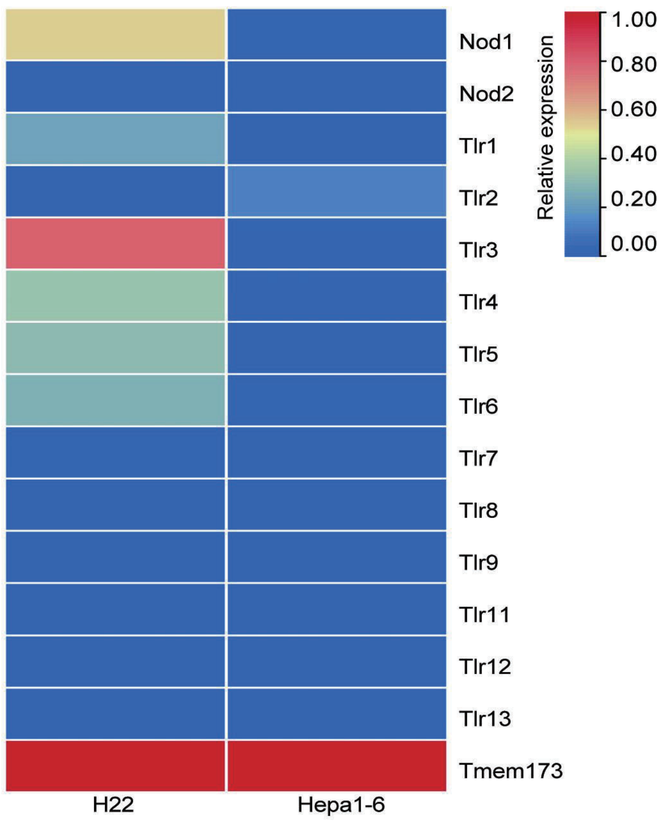

B

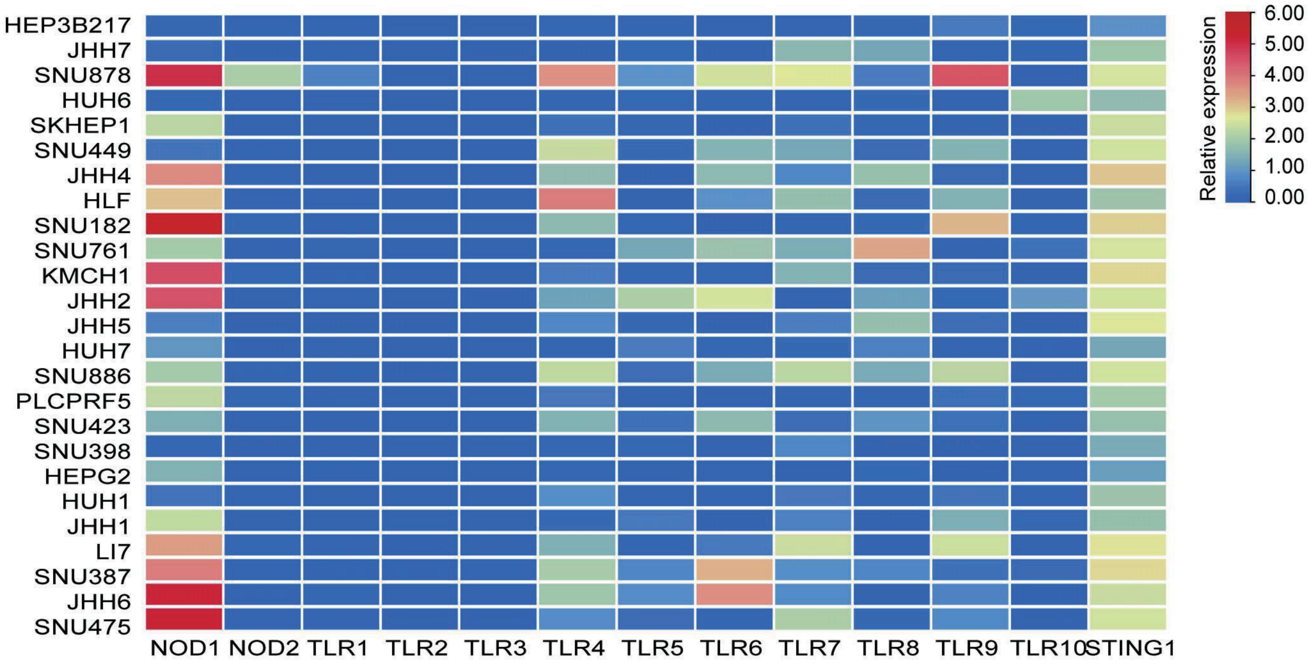

Supplementary Figure 4

A

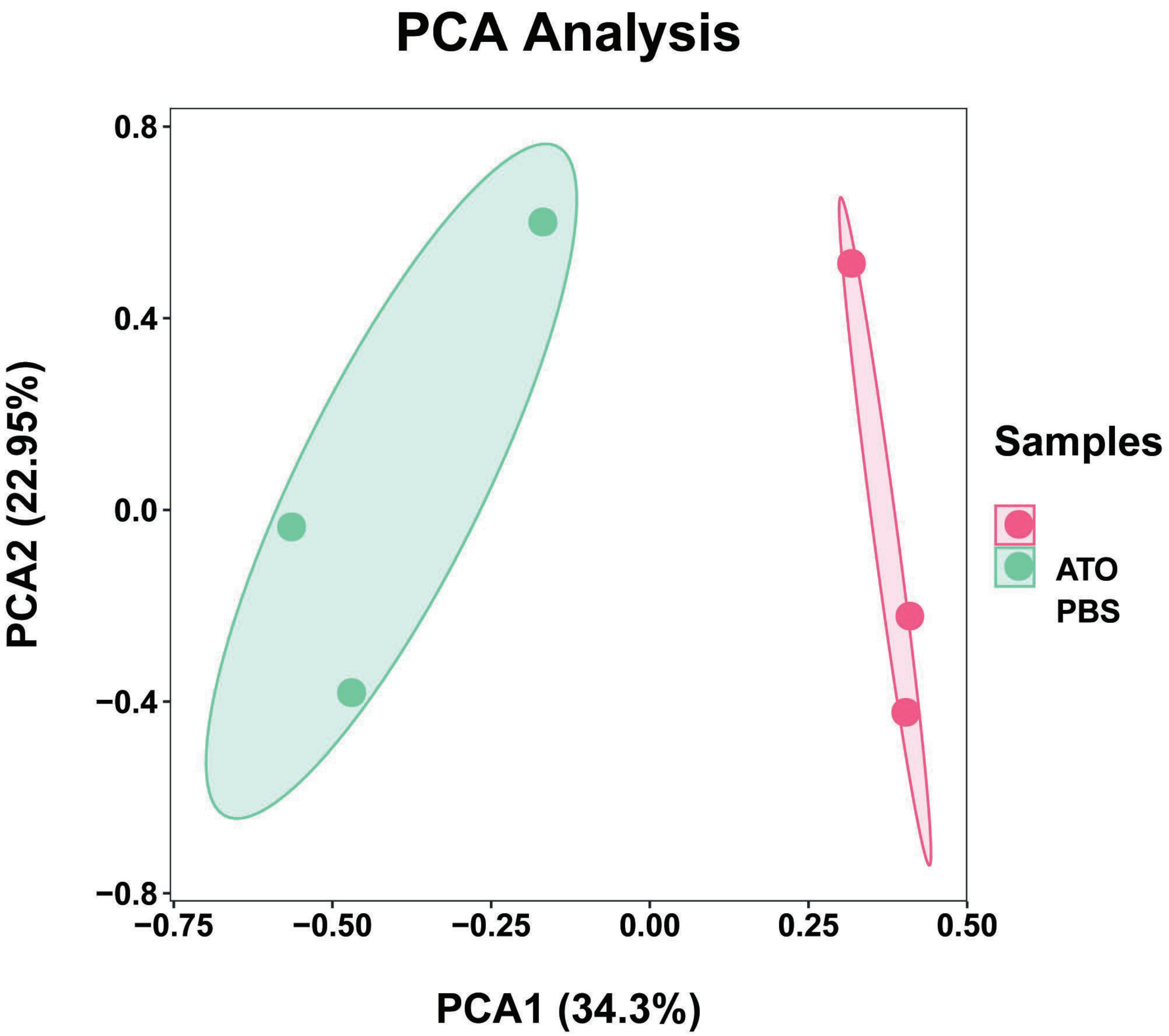

Supplementary Figure 5

A

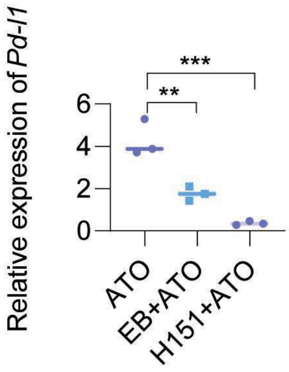

B

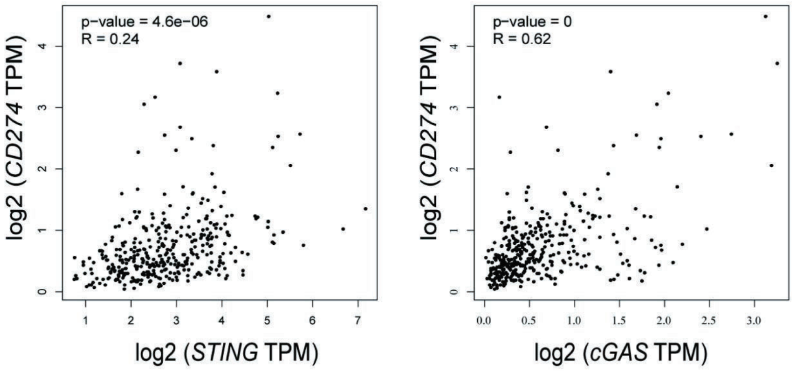

Supplementary Figure 6

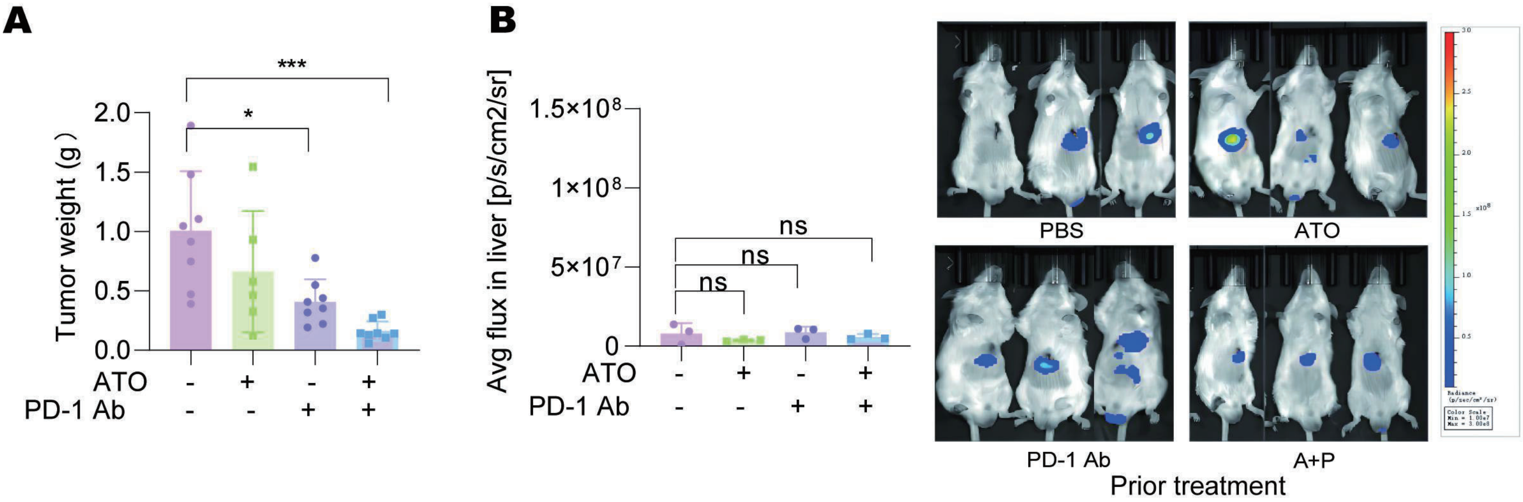

Supplementary Figure 7

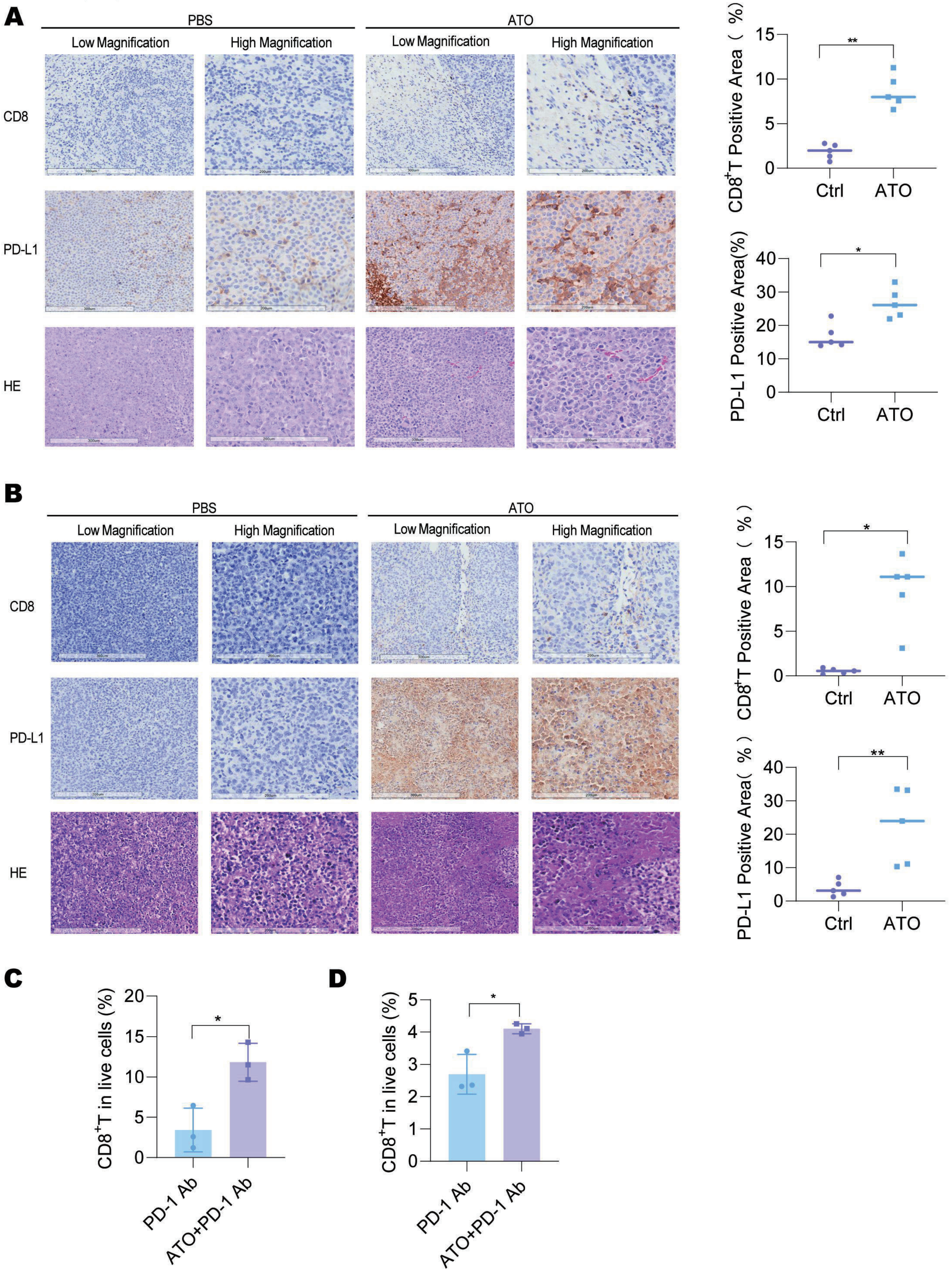

Supplementary Figure 8

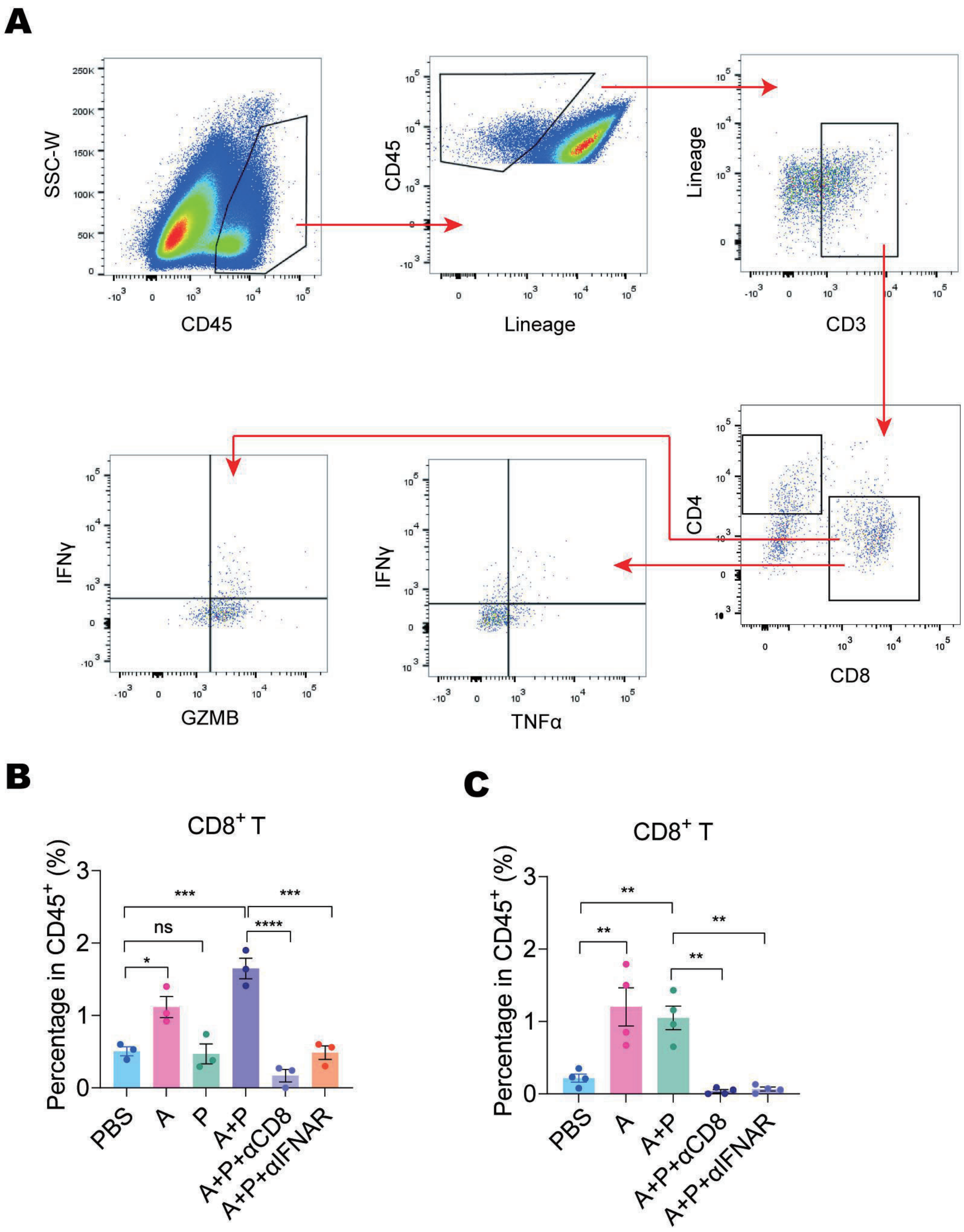

Supplementary Tables

Supplementary Table 4. Primer sequence list

Primer	Sequence (5' to 3')
mCd274-F	TGCGGACTACAAGCGAATCACG
mCd274-R	CTCAGCTTCTGGATAACCCTCG
mCxcl10-F	ATCATCCCTGCGAGCCTATCCT
mCxcl10-R	GACCTTTTTTGGCTAAACGCTTTC
mCxcl11-F	CCGAGTAACGGCTGCGACAAAG
mCxcl11-R	CCTGCATTATGAGGCGAGCTTG
mCxcl12-F	GGAGGATAGATGTGCTCTGGAAC
mCxcl12-R	AGTGAGGATGGAGACCGTGGTG
mCxcl9-F	CCTAGTGATAAGGAATGCACGATG
mCxcl9-R	CTAGGCAGGTTTGATCTCCGTTC
mIfnb1-F	GCCTTTGCCATCCAAGAGATGC
mIfnb1-R	ACACTGTCTGCTGGTGGAGTTC
mIfng-F	CAGCAACAGCAAGGCGAAAAAGG
mIfng-R	TTTCCGCTTCCTGAGGCTGGAT
mH2db-F	AGTGGTGCTGCAGAGCATTACAA
mH2db-R	GGTGACTTCACCTTTAGATCTGGG
mTnf-F	GGTGCCTATGTCTCAGCCTCTT
mTnf-R	GCCATAGAACTGATGAGAGGGAG

mIL1b-F	TGGACCTTCCAGGATGAGGACA
mIL1b-R	GTTCATCTCGGAGCCTGTAGTG
18s-F	CGGCTACCACATCCAAGGAA
18s-R	GCTGGAATTACCGCGGCT
D-loop3-F	TCCTCCGTGAAACCAACAA
D-loop3-R	AGCGAGAAGAGGGGCATT
Nd1-F	CTAGCAGAAACAAACCGGGC
Nd1-R	CCGGCTGCGTATTCTACGTT
Gapdh-F	AGGCCGGTGCTGAGTATGTC
Gapdh-R	TGCCTGCTTCACCACCTTCT

Supplementary Table 5. Antibody catalog

Antibody	Manufactor	Category number
p-STING	Cell signaling	S365
STING	Cell signaling	D1V5L
p-TBK1/NAK	Cell signaling	S172
TBK1/NAK	Cell signaling	D1B4
p-IRF-3	Cell signaling	S396
IRF-3	Cell signaling	D83B9
β-Actin	Proteintech	81115-1-RR
GAPDH	Proteintech	60004-1-Ig

p-p65	Cell signaling	3033
p65	Cell signaling	8242
p-p38	Cell signaling	4511
p38	Cell signaling	8690
p-AKT	Cell signaling	4060
AKT	Cell signaling	9272
LC3A/B	Cell signaling	4108
P62	Cell signaling	5114
BECLIN-1	Cell signaling	3738
$CD3^+T$	Invitrogen	2423799
$CD4^+T$	Invitrogen	2403342
$CD8^+T$	Biolegend	100722
CD8α	Cell signaling	98941
PD-L1	Cell signaling	13684
$CD8^+T$	Selleck	A2102
IFNAR1	Selleck	A2121
IFN-γ	Biolegend	505809
TNF-α	Biolegend	506305
GZMB	Biolegend	515408
PD-1	BioXcell	BE0146

Supplementary Table 6. Inhibitor list

Inhibitor	Manufactor	Category number
Necrostatin 1	Selleck	S8037
Ferrostatin 1	Selleck	S7243
Chloroquine	Selleck	S6999
Z-VAD (OMe)-FMK	Selleck	S7023
N-acetyl-L-cysteine	Selleck	S1623
H151	MedChemExpress	HY-112693
