# Supplementary material for: Arsenic trioxide augments immunogenic cell death and induces cGAS-STING-IFN pathway activation in hepatocellular carcinoma
Source: Cell Death Dis. 2024 Apr 29;15(4):300. doi: 10.1038/s41419-024-06685-8 (PMC11058202; doi:10.1038/s41419-024-06685-8)

Figure 5D

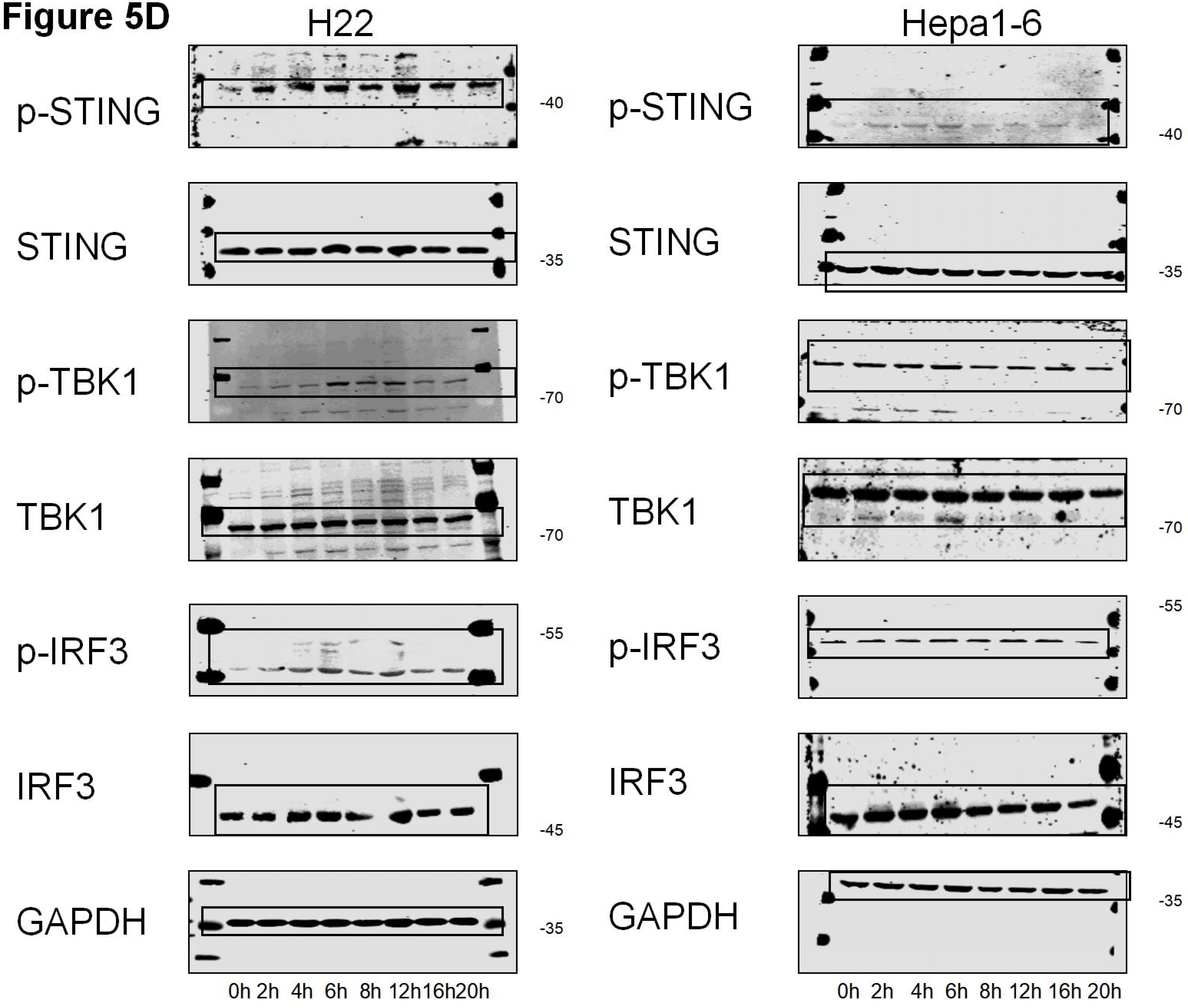

Figure 5F

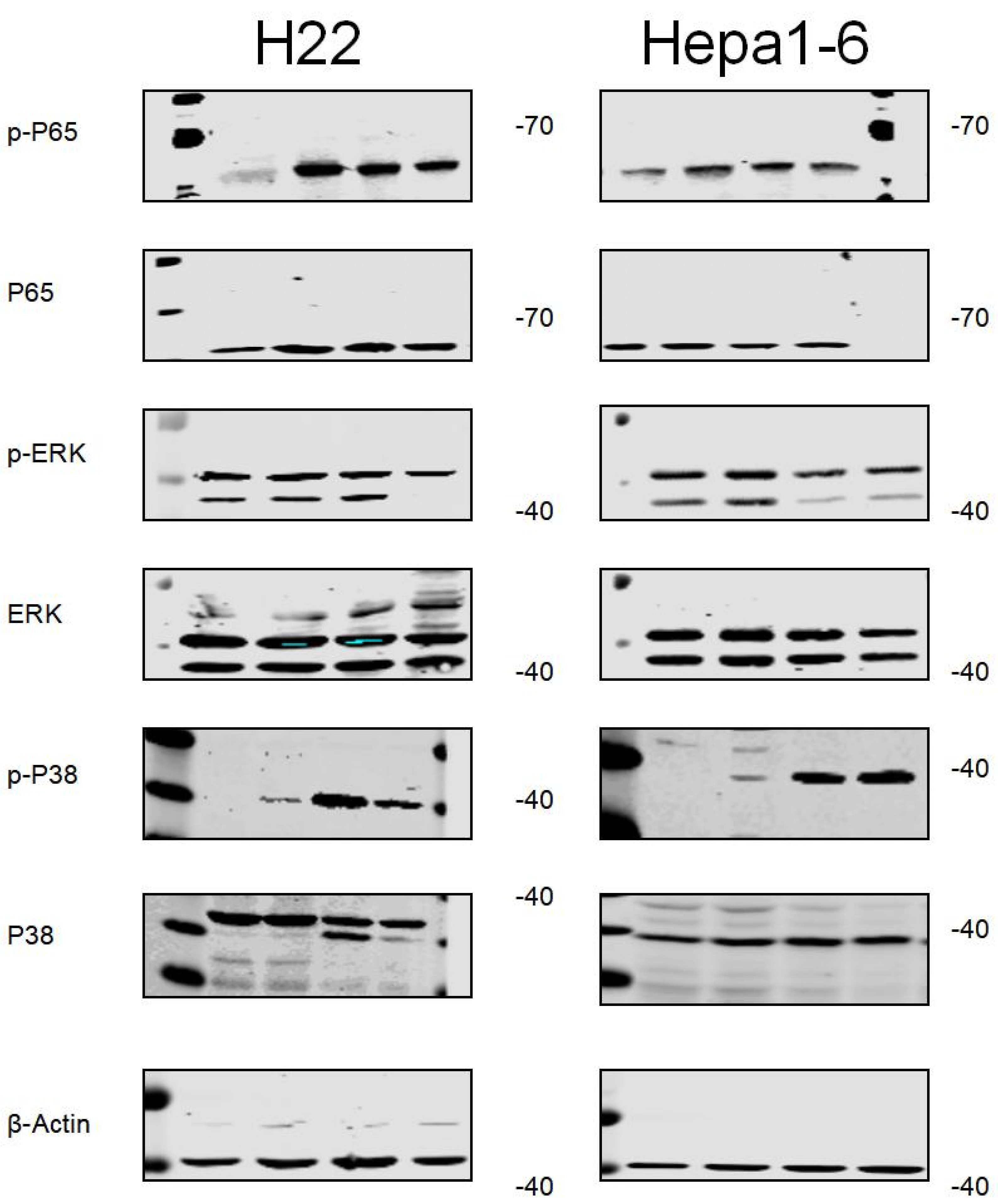

Figure 6C

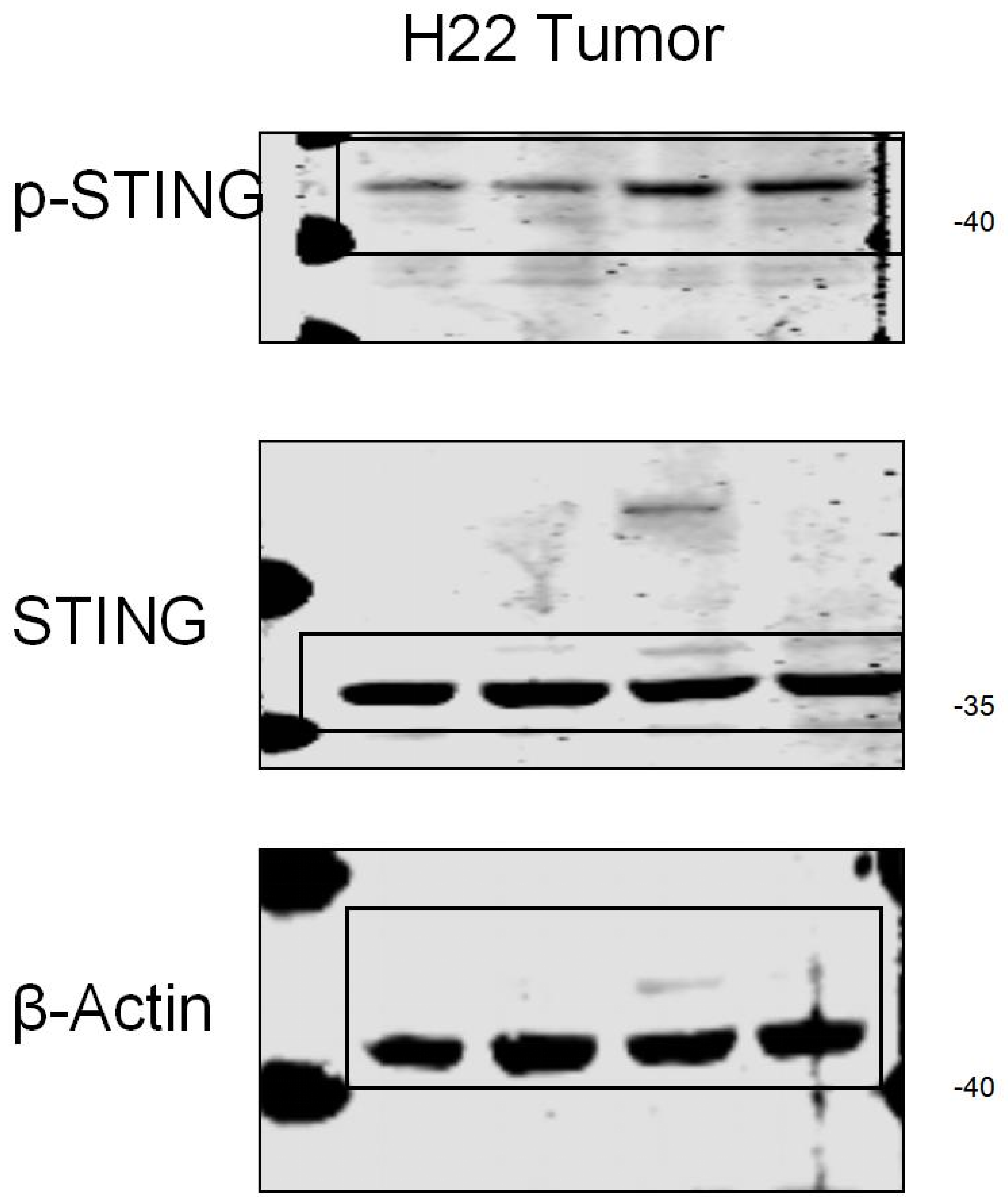

Supplementary Figure 2B

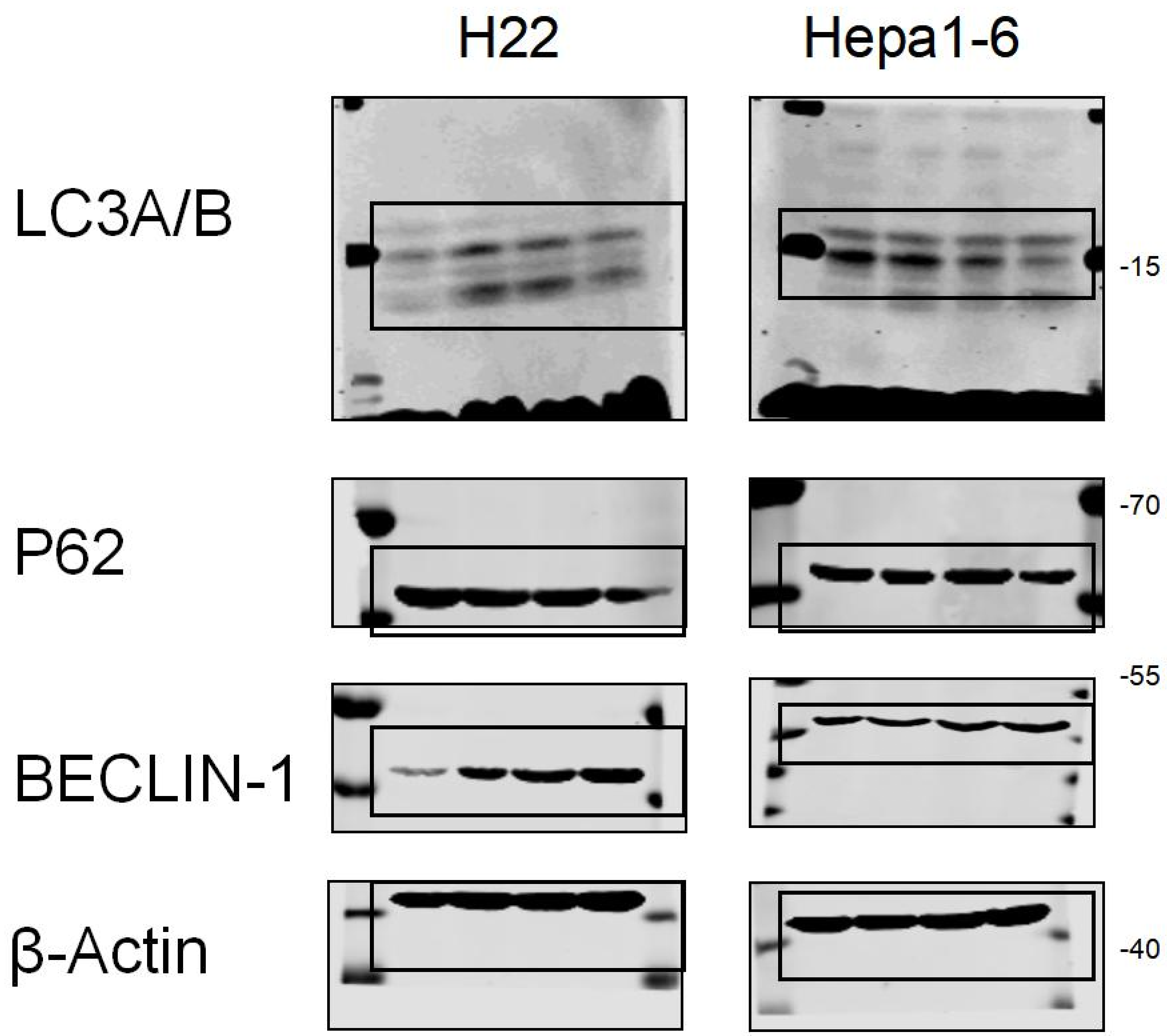

Supplementary Figure 2C

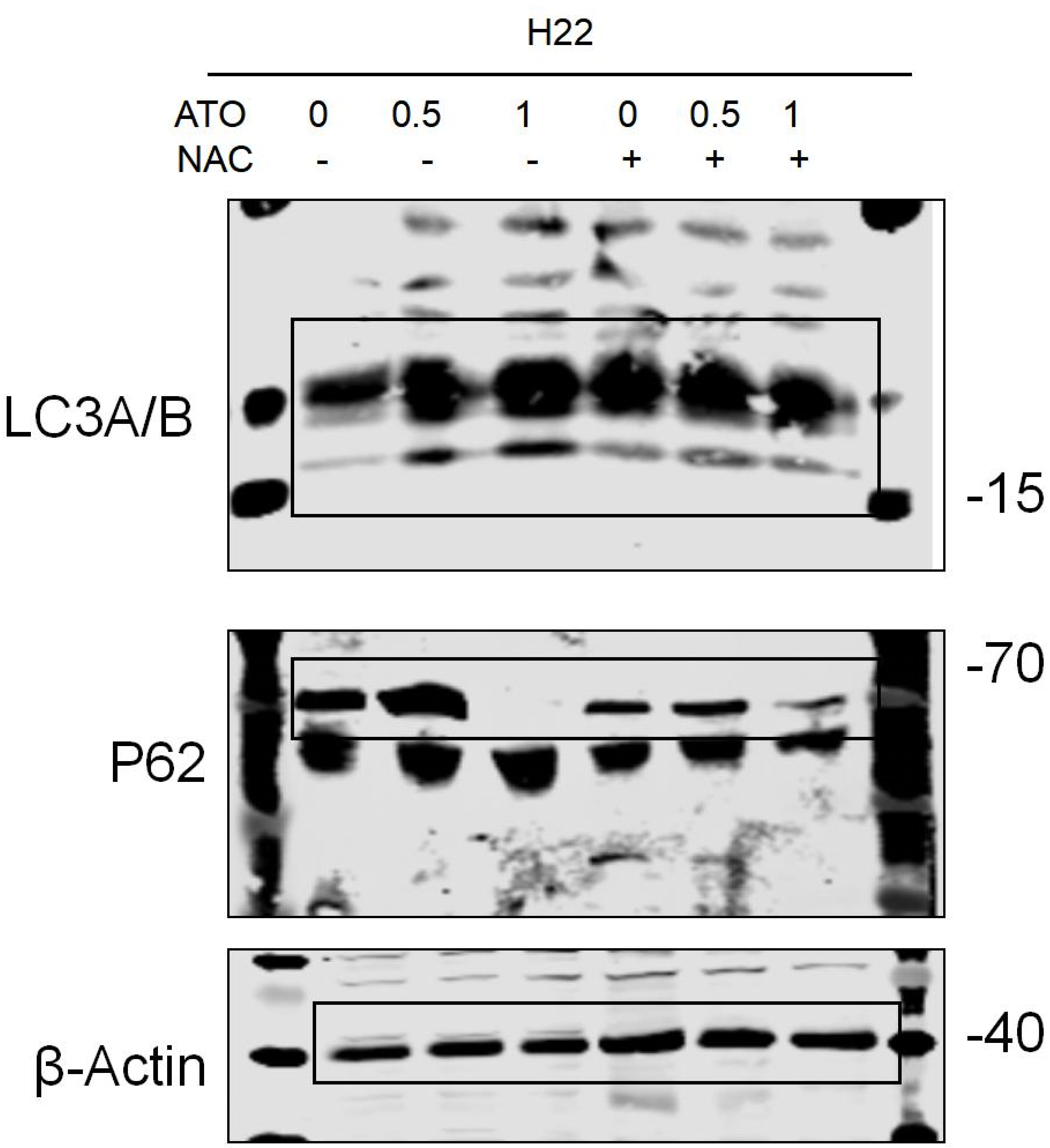

Supplementary Figure 2D

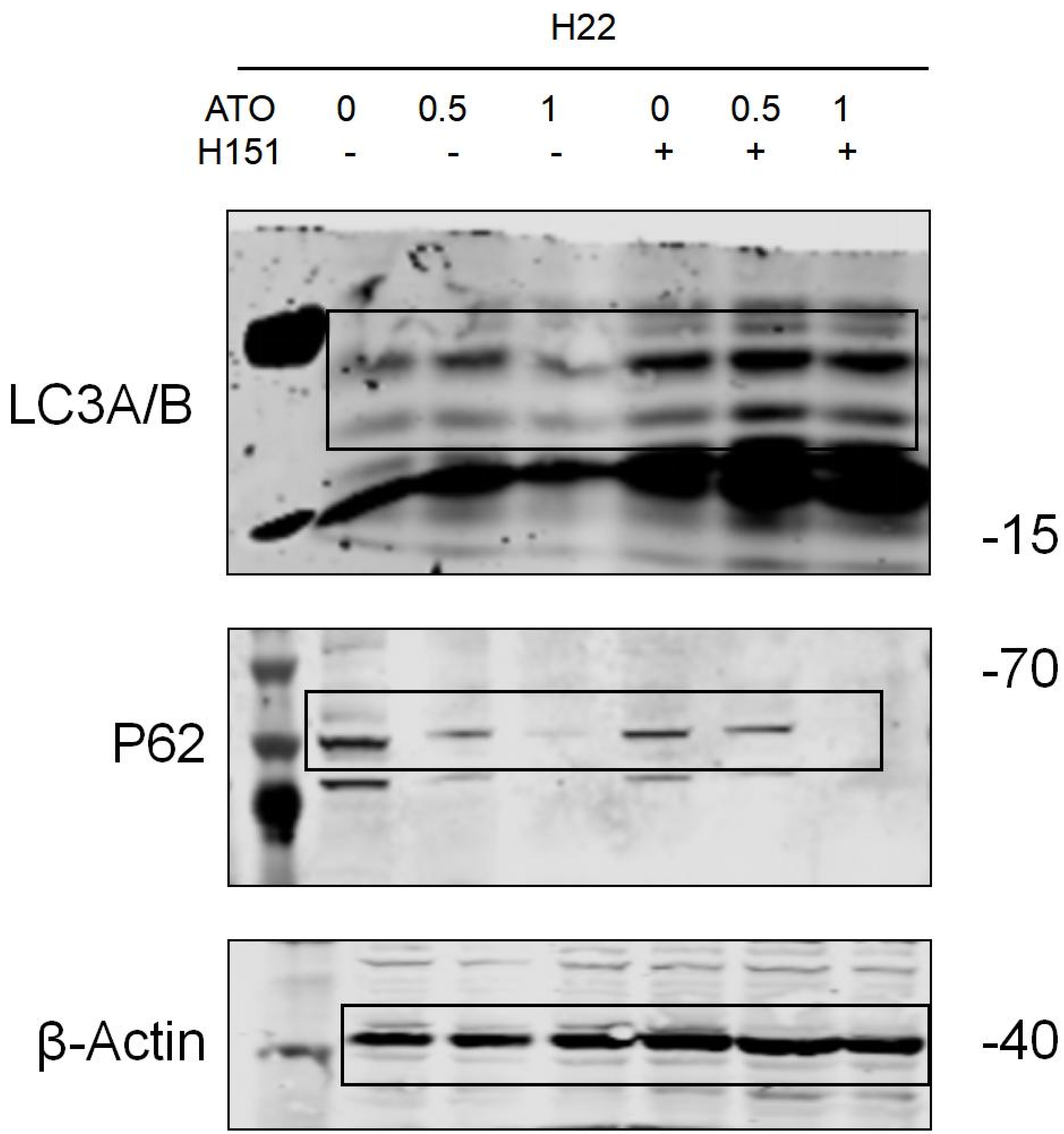

Supplement: Supplementary file 3 — Full and uncropped Western blots [file 41419_2024_6685_MOESM3_ESM.pdf]
